# Implementation of a decision aid to promote shared decision-making on mode of birth in low-risk pregnant women: a cross-sectional study within the QUALI-DEC hybrid trial

**DOI:** 10.1136/bmjgh-2025-022365

**Published:** 2026-03-03

**Authors:** Truc Phuong Nguyen, Ana Pilar Betran, Guillermo Carroli, Charles Kaboré, Pisake Lumbiganon, Quoc Nhu Hung Mac, Celina Gialdini, Camille Etcheverry, Barbara Vololonarivelo, Kristi Sidney Annerstedt, Ramón Escuriet, Claudia Hanson, Allison Shorten, Alexandre Dumont, Consortium of QUALI-DEC Project, Alexandre Dumont

**Affiliations:** 1 Ceped, IRD, Université Paris Cité, Paris, France; 2 Reproductive Health and Research, World Health Organization, Geneva, Switzerland; 3 Centro Rosarino de Estudios Perinatales, Rosario, Argentina; 4 Institut de Recherche en Sciences de la Sante, Ouagadougou, Burkina Faso; 5 Obstetrics and Gynaecology, Khon Kaen University Faculty of Medicine, Khon Kaen, Thailand; 6 Department of Obstetrics and Gynecology, Pham Ngoc Thach University of Medicine, Ho Chi Minh, Viet Nam; 7 Facultat de Ciències de la Salut Blanquerna, Universitat Ramon Llull, Barcelona, Spain; 8 Global Public Health, Karolinska Institutet, Stockholm, Sweden; 9 Health Care Division, Government of Catalonia Catalan Health Service, Barcelona, Spain; 10 Faculty of Health Sciences, Fundacio Blanquerna, Barcelona, Spain; 11 London School of Hygiene and Tropical Medicine, London, UK; 12 Nursing, The University of Alabama, Birmingham, Alabama, USA

**Keywords:** Decision Making, Maternal health, Public Health, Epidemiology

## Abstract

**Introduction:**

Implementing shared decision-making (SDM) in maternity care remains challenging in low-income and middle-income countries (LMICs). Decision aids can support SDM, but evidence on their effectiveness in such settings is limited. We assessed the impact of a decision analysis tool (DAT) for pregnant women on mode of birth (MOB) within the QUALIty DECision-making project, a multisite, multicountry pragmatic trial to reduce unnecessary caesarean sections.

**Methods:**

We conducted a cross-sectional survey among postpartum women considered at low risk for caesarean section in early pregnancy and who delivered in 32 hospitals across Argentina, Burkina Faso, Thailand and Viet Nam. Associations between DAT exposure and selected outcomes were analysed using multilevel, multivariate regression models adjusting for confounders and cluster effects.

**Results:**

Of 2368 women included, 249 (11%) had used it outside antenatal care visits, 212 (9%) had heard of but not used it, and 1907 (80%) had never heard of the DAT. Compared with women who had never heard of the DAT, users were more likely to identify at least three risks/benefits of each MOB (adjusted OR (aOR) 1.9; 95% CI 1.3 to 2.8; p=0.001) and to communicate their preferred MOB to providers (aOR 2.3; 95% CI 1.5 to 3.6; p<0.001). DAT users were less likely to prefer caesarean section in late pregnancy (aOR 0.4; 95% CI 0.2 to 0.8; p=0.006) and reported higher birth experience and satisfaction scores (adjusted β=1.9; 95% CI 0.5 to 3.3; p=0.006).

**Conclusions:**

The use of the DAT was associated with improved knowledge, communication of birth preferences, lower caesarean preference and greater satisfaction, without adverse outcomes. Findings suggest that decision aids can strengthen SDM and promote respectful, women-centred maternity care in LMICs.

**Trial registration number:**

ISRCTN67214403

**WHAT IS ALREADY KNOWN ON THIS TOPIC**

- Shared decision-making implementation in maternity care remains challenging in most low-income and middle-income countries. Decision aids can support shared decision-making, but evidence on their effectiveness remains limited in such settings.

**WHAT THIS STUDY ADDS**

- The use of the decision analysis tool (DAT) was associated with significant differences in maternal outcomes related to quality of decision, quality of decision-making process, care behaviours and satisfaction with care.

**HOW THIS STUDY MIGHT AFFECT RESEARCH, PRACTICE OR POLICY**

- Future research will assess the effects of the combination of the DAT with other interventions targeting health care providers on medical practice and perinatal outcomes.

## Introduction

 Patient-centred care is widely recognised as a cornerstone of high-quality healthcare, emphasising respect for patients’ values, preferences and needs—particularly through shared decision-making^1^ (SDM). Research has shown that patients who actively participate in their care decisions are more likely to experience better health outcomes and greater satisfaction.^2 3^ In maternity care, empowering women to engage in decision-making is increasingly recognised as a fundamental component of respectful and high-quality care.^4^ However, many women lack adequate support and resources to make informed, value-based decisions about their childbirth options.^5^

Patient decision aids are evidence-based tools designed to help patients make specific and deliberated choices among healthcare options.^6^ They include at least information about options and outcomes and also provide guidance in the decision-making steps or explicit methods to clarify values.^7^ Studies from high-income countries indicate that decision aids for pregnant women around childbirth can enhance their knowledge and autonomy, reduce decisional conflict, promote physiological birth preferences and improve childbirth experiences and health outcomes.^58^,^10^

However, evidence on their effectiveness remains limited in low-income and middle-income countries (LMICs),^10^ where processes of SDM need to be strengthened.^11^ Previous studies have shown that pregnant women are not sufficiently involved in decision-making, even when they have clear preferences.^12 13^ In the context of increasing use of caesarean sections (CSs) rates, substantial gaps exist between women’s preferences and actual delivery modes. This mismatch raises questions about women’s involvement in the decision-making processes surrounding the mode of birth (MOB). Moreover, the majority of women prefer vaginal delivery and rising CS rates in LMICs are unlikely to be mainly driven by women’s demand for CS.^14^

### QUALIty DECision-making project

In 2020, we started the *Appropriate use of CS through QUALIty DECision-making by women and providers* (QUALI-DEC) project to implement an evidence-based multifaceted non-clinical intervention in four LMICs: Argentina, Burkina Faso, Thailand and Viet Nam. This intervention simultaneously targeted pregnant women and healthcare providers (HCPs) to improve decision-making on MOB and combined four components: (1) opinion leaders to improve evidence-based clinical practices; (2) audits and feedback to help HCPs identify unnecessary CS; (3) implementation of companionship during labour and childbirth to support labouring women and (4) a *decision-analysis tool* (DAT) to help women make an informed decision on MOB.^15^

### The DAT

The DAT was designed for low-risk pregnant women and provides information about risks and benefits of both MOB, as well as facilitating the clarification of their personal values. The DAT was available to women either as a printed booklet or as a mobile application developed for the QUALI-DEC project; its full content and development process are described in detail elsewhere.^16^ Low-risk women are those who are pregnant with a single fetus and no previous CS. This tool was adapted from the decision aid ‘*Birth Choices. What is best for you… Vaginal or Caesarean Birth?’*^17^ and meets the criteria of the International Patient Decision Aid Standards.^7^ The DAT has two sections: the first provides evidence-based information on the risks and benefits of each MOB in an engaging, appropriate plain language for women; the second guides women to reflect on what matters most to them, thereby preparing them for meaningful discussions with HCPs. We hypothesised that the DAT would benefit low-risk women regardless of whether they preferred a vaginal birth or a CS, as it promotes meaningful dialogue with HCPs. Such conversations may help address factors influencing caesarean preferences while also supporting women who prefer vaginal birth in realising their desired mode of delivery. This study is nested within a pragmatic hybrid effectiveness-implementation type III trial, which includes an effectiveness and a process evaluation of the multi-faceted intervention in Argentina, Burkina Faso, Thailand and Viet Nam.^18^ The aim of this study is to evaluate the impact of the DAT on the SDM process and outcomes regarding the MOB for low-risk pregnant women.

## Methods

### Study design and setting

Patients or the public were not involved in the design, or conduct, or reporting, or dissemination plans of our research. The protocol for the QUALI-DEC project has been published elsewhere.^15^ Briefly, 32 hospitals (eight in each country) were selected for the intervention implementation, based on policymakers’ concerns and commitment to reducing unnecessary CS rates in their context. Two cross-sectional postpartum surveys were conducted in each country to assess differences between before and after the intervention. This study is an ancillary analysis of the second cross-sectional postpartum survey which was conducted from 16 October–31 October 2023 in Burkina Faso, 18 March–14 July 2024 in Thailand, 22 April–14 May 2024 in Viet Nam and 24 June–16 September 2024 in Argentina.

### Participants and sample size

The detailed description of the QUALI-DEC cross-sectional survey has been published elsewhere^19^ (see description in online supplemental information text S1). Briefly, postpartum women were eligible for the interview if they had given birth to a live-born child beyond 22 weeks of gestation (28 weeks in Burkina Faso). Women were excluded if they had a major health problem following childbirth, according to the judgement of HCPs, gave birth to a stillborn child, experienced neonatal death, or if their newborns presented severe morbidity. Women who delivered at home or in another health facility (postnatal transfer) were also excluded. The survey included all eligible women in hospitals with fewer than ten deliveries per day, while a random sample of women was used in facilities with 10 or more deliveries per day.

The sample size estimate was not calculated specifically for this ancillary study but for the trial effectiveness evaluation. The calculation was based on the expected before-after difference in women’s birth experience and satisfaction (BES) scores, that is, 470 women per country.^15^ The minimum number of women to approach during recruitment was 564 women in each country (71 women per hospital), assuming a 10% non-response rate and 10% ineligible women.

### Outcomes

Proxy indicators for SDM were defined according to the four objectives of a DAT, according to the Ottawa Decision Support Framework^20^: (1) quality of decision (three or more evidence-based risks and benefits identified of any MOB), (2) quality of decision-making process (preferred MOB told during antenatal care or at admission), (3) care behaviours (having a preferred MOB at late pregnancy, preference for CS, trial of labour, vaginal birth, matched preferred and planned MOB, matched preferred and actual MOB) and (4) health outcomes (postpartum haemorrhage treatment, neonatal intensive care unit admission, breastfeeding during hospital stay, BES score with intrapartum care) (detail outcomes description in online supplemental table S1 and S2).

Information on sociodemographic characteristics, preferred MOB, identified evidence-based risks and benefits of each MOB and BES score was collected during the postpartum period, before hospital discharge, by a trained social scientist. Clinical information on pregnancy, labour and delivery management, maternal and child health outcomes was extracted from medical records by trained clinical data collectors. Both pieces of information were collected via standardised forms.

### Exposure

Participants’ exposure to the DAT was determined based on their answers to two consecutive interview questions. While asking these questions during the interview, the data collectors showed the women both versions of the tool (the printed booklet and the app on a smartphone) to help them recognise it: (1) ‘Have you heard or seen this booklet or App?’ and (2) ‘Have you ever used this tool outside of your antenatal consultation?’. The exposures were categorised into three groups: (1) ‘Used’ when a woman reported using the DAT outside of her antenatal care visit, regardless of the format; (2) ‘Only heard’ when she heard about the DAT but never used it; and (3) ‘Never heard’ when she had neither heard nor seen the DAT during pregnancy.

Two formats of the DAT were made available to women in each participating country: a booklet and a mobile application (App). The printed booklet was given to women at the discretion of HCPs during antenatal care visits or was made freely accessible in hospital waiting areas. Its content was culturally adapted for each participating country, resulting in variations in presentation: 16 pages in Argentina, 18 pages in both Burkina Faso and Thailand, and a more concise 4–6 pages in Viet Nam. The App could be accessed by scanning a QR code displayed on posters promoting the QUALI-DEC project, placed prominently in hospital lobbies. To ensure accessibility, the App was developed in six languages: Burmese, English, French, Spanish, Thai and Vietnamese, matching the linguistic needs of the participating countries. Furthermore, multimedia formats of the DAT, including videos and podcasts, were displayed on screens in antenatal clinic waiting rooms to ensure that pregnant women were aware of the DAT. The availability of these materials varied depending on the resources and infrastructure of each participating hospital. In addition, short promotional videos (reels) were disseminated through social media platforms such as Instagram, TikTok and YouTube to further expand awareness and encourage the uptake of the DAT, particularly in Argentina, Thailand and Viet Nam.

### Potential confounders

Sociodemographic, pregnancy-related and organisational factors were selected based on existing literature and contextual relevance. Sociodemographic factors included country of residence, marital status, maternal age, level of education, maternal occupation, partner’s occupation, having a smartphone and household wealth index. The wealth index is a context-specific composite index, developed through variable selection and component analysis carried out in collaboration with local investigators. Pregnancy-related factors include any preference for MOB at early pregnancy; frequency and place of antenatal care visits; referral from another hospital; any complications during pregnancy including pre-labour rupture of membrane, gestational hypertension, chronic hypertension, pre-eclampsia, eclampsia, cardiac/renal disease, chronic respiratory conditions, suspected foetal growth impairment, diabetes, vaginal bleeding during second Half of pregnancy, condyloma acuminatum, confirmed HIV/AIDS, cholestasis; and other obstetric or medical conditions that could be an indication for a CS. Organisational factors include women’s accessibility to healthcare services which was estimated by the time to get to the hospital (in minutes), status of the maternity unit where the woman delivered (academic or not, reference level, totally public or private practice for all/some doctors) and the implementation score of the paper-based DAT at hospital level. This score was calculated based on two items: (a) whether the booklet was available in waiting areas, antenatal clinics, doctors’, nurses’ stations or other locations; and (b) whether the booklet was administered to eligible pregnant participants. Each item was scored as either 1 or 0 (1: yes; 0: no). The information on the implementation score was collected during regular monitoring visits and consultations with implementing partners.^18^

### Quantitative variables

Age, parity, antenatal care attendance, hospital accessibility, wealth index and DAT implementation score at hospital level were treated as categorical variables, based on distribution and theoretical relevance (eg, age grouping as <25, 25–35, >35; parity as nulliparous versus multiparous, wealth index as higher, intermediate, lower; time to reach the hospital as less than 30 min vs 30 min and more). Healthcare facilities were categorised as having a high level of implementation if the score was higher than 75%. BES scores were treated as continuous variables.

### Analysis

While the QUALI-DEC trial evaluates the strategy as a whole, the present analysis focuses specifically on exploring the associations between DAT exposure and selected outcomes. The analysis was restricted to low-risk women who were eligible for the use of DAT^16^: women with a singleton pregnancy and having never given birth by CS in a previous pregnancy. In this study, ‘low-risk women’ refers to those classified as having a low baseline probability of caesarean delivery early in pregnancy. Since the DAT was integrated into routine antenatal care and could be administered from the first visit, before later-pregnancy clinical information (such as foetal presentation) was available. We restricted eligibility to criteria measurable during early pregnancy. This approach ensured alignment to the population for whom reducing unnecessary primary CS is most relevant.

All variables described in the outcomes, potential confounders and quantitative variables subsections were included in our analysis to build up multilevel multivariate regression models.

Descriptive statistics were used to summarise participant characteristics and key variables. Categorical variables are presented as frequencies and percentages, while continuous variables are summarised as medians and IQRs. Women’s characteristics were initially compared between exposure groups via χ² tests and crude associations between DAT exposure and each of the 16 outcomes were tested using χ^2^ tests for categorical variables and the Kruskal-Wallis test for continuous variables.

To control for confounders, we used multivariable logistic and linear mixed-effects regression models, as appropriate. A stepwise model selection procedure based on the Akaike Information Criterion was employed to identify the most parsimonious models. To account for clustering at the facility level, a random intercept for the variable hospital (n=32) was included, capturing site-specific differences in implementation and clinical practices.

During data collection, some responses were classified as 'unknown’ when the information provided was unclear. These were later treated as missing data during analysis. No data imputation was performed. The dataset had less than 3.5% missing values, except for the partner occupation, which had 6.5% of missing values.

A sensitivity analysis was conducted to evaluate the robustness of the findings by excluding women giving birth in Burkina Faso where the number of DAT users was low (n=8).

The datasets analysed during the current study are publicly available in Zenodo.^21^,^24^

All statistical analyses were performed using R software (V.4.4.2) in RStudio (V.2024.12.1+563).

## Results

From a total of 5830 women who gave birth during the study period, 3545 were randomly selected and 3071 of whom provided consent (online supplemental figure S1). As per the analysis plan, we further excluded 703 women due to multiple pregnancies and prior CS. We then analysed 2368 women who were eligible for the DAT.

### Exposure and women’s acceptability

Among them, 1907 (80%) women had never heard of the DAT, 212 (9%) had heard of the DAT but never used it, and 249 (11%) women had used the DAT outside antenatal care visits. Among the 461 women who either used or only heard about the DAT, 370 (80%) reported receiving it from their HCPs. Among all 461 women, 94 (20%) received the DAT in the first trimester, 79 (17%) in the second trimester and 202 (44%) in the third trimester of pregnancy. The remaining 86 women (19%) included 12 who were unsure of the timing and 74 who reported awareness of the DAT but did not receive a personal introduction to the tool. Among the 249 women who reported using the DAT, 209 (84%) used the paper-based format, while 13 (5%) used the App, and 28 (11%) used both formats. Among users, 122 (49%) used the DAT once, 69 (28%) used it twice, and 58 (23%) used it three times or more, and 72 (29%) reported writing their motivation into the tool. A total of 245 (98%) users found the DAT easy to use, 228 (92%) users agreed that the DAT facilitated communication with HCPs, and 233 (94%) users agreed that it helped them clarify their birth preference. A total of 242 (97%) users agreed or strongly agreed that they would recommend the DAT to other pregnant women.

### Participant characteristics

The distribution of sociodemographic characteristics varied across the three exposure groups (table 1). Women aged ≥35 years were more likely to be DAT users, whereas women aged 25–34 years were more represented in the Only-heard group. While most women lived in urban areas, rural women were proportionally more represented among those who had only heard about the DAT. Women with secondary education were the majority across groups, but the proportion of women with tertiary education was highest among DAT users, suggesting a gradient favouring DAT use with increasing education. Regarding socioeconomic status, both women in the lowest and highest wealth index categories seem more likely to use the DAT, while those in the middle category were less likely to do so. Most pregnancy-related and organisational factors were also associated with exposure (table 2), particularly attending ANC in participating hospitals and giving birth in facilities with higher levels of DAT implementation.

**Table 1:** Sociodemographic characteristics of participants, by exposure group* N=2368 women†

Characteristic	Used **N=249** **N (%)**	Only heard **N=212** **N (%)**	Never Heard **N=1907** **N (%)**	P value
Country of residence				<0.001
Argentina	111 (45)	53 (25)	310 (16)	
Burkina Faso	7 (2.8)	21 (9.9)	721 (38)	
Thailand	38 (15)	36 (17)	461 (24)	
Viet Nam	93 (37)	102 (48)	415 (22)	
Marital status				0.2
Married/living with a partner	243 (98)	206 (98)	1752 (96)	
Separated/single/widow	5 (2)	4 (1.9)	65 (3.6)	
Maternal age				0.036
<25	83 (33)	60 (28)	719 (38)	
25–34	123 (49)	122 (58)	912 (48)	
≥35	43 (17)	30 (14)	276 (14)	
Education level				0.041
Secondary or lower	167 (67)	154 (73)	1421 (75)	
Tertiary education	82 (33)	58 (27)	486 (25)	
Geographic context				<0.001
Rural	64 (26)	71 (33)	411 (22)	
Urban	185 (74)	141 (67)	1496 (78)	
Maternal occupation				<0.001
Employed formal sector	76 (31)	61 (29)	371 (19)	
Employed informal sector	75 (30)	81 (38)	721 (38)	
Unemployed/housewife	98 (39)	70 (33)	814 (43)	
Partner’s occupation				0.013
Employed formal sector	91 (40)	66 (33)	578 (32)	
Employed informal sector	128 (57)	122 (60)	1148 (64)	
Unemployed/homemaker	7 (3)	14 (7)	59 (4)	
Wealth index				<0.001
Higher	99 (40)	77 (36)	611 (32)	
Intermediate	30 (12)	37 (17)	437 (23)	
Lower	120 (48)	98 (46)	858 (45)	
Having a smartphone	246 (99)	207 (98)	1861 (98)	0.6

* Used: women reported using the DAT outside of their antenatal care visit; Only heard: women heard about the DAT but never used it; Never heard: women had neither heard nor seen the DAT during pregnancy.

† Women with single pregnancy and without prior caesarean section.

DAT, decision analysis tool.

**Table 2:** Pregnancy-related and organisational factors, by exposure group* N=2368 women†

Characteristic	Used **N=249** **N (%)**	Only heard **N=212** **N (%)**	Never heard **N=1907** **N (%)**	P value
Parity				0.2
Nulliparous	145 (58)	106 (50)	1008 (53)	
Multiparous	104 (42)	106 (50)	896 (47)	
Preference at early pregnancy				0.004
Caesarean section	14 (6)	9 (4)	92 (5)	
Vaginal birth	209 (84)	180 (85)	1471 (77)	
No preference	26 (10)	23 (11)	343 (18)	
Having complication during pregnancy‡				0.14
Yes	92 (37)	75 (36)	785 (41)	
No	157 (63)	136 (64)	1116 (59)	
Referred from another hospital				<0.001
Yes	10 (4)	20 (9)	600 (31)	
No	239 (96)	192 (91)	1307 (69)	
Number of antenatal care visits				<0.001
4 visits or more	236 (97)	191 (93)	1582 (86)	
Less than 4 visits	7 (3)	14 (6.8)	264 (14)	
Antenatal care visit in participating hospital				<0.001
Yes	184 (74)	134 (63)	731 (38)	
No	65 (26)	78 (37)	1176 (62)	
Antenatal care visit in another private facility, outside participating hospital				0.035
Yes	76 (31)	87 (41)	627 (33)	
No	173 (69)	125 (59)	1280 (67)	
Time to get to hospital				<0.001
Less than 30 min	167 (67)	122 (58)	914 (49)	
30 min or more	82 (33)	89 (42)	948 (51)	
Referral level				0.8
Tertiary	146 (59)	123 (58)	1142 (60)	
Primary–secondary	103 (41)	89 (42)	765 (40)	
Teaching facility				0.004
Yes	187 (75)	133 (63)	1790 (73)	
No	62 (25)	79 (37)	517 (27)	
Any private practice in the maternity unit				<0.001
Completely (private hospital)	11 (4)	26 (13)	82 (4)	
Partially (for some providers)	106 (43)	94 (44)	873 (46)	
No	132 (53)	92 (43)	952 (50)	
Level of implementation of the paper-based decision aid				<0.001
High	229 (92)	170 (80)	1563 (82)	
Low	20 (8)	42 (20)	344 (18)	

* Used: women reported using the DAT outside of their antenatal care visit; Only heard: women heard about the DAT but never used it; Never heard: women had neither heard nor seen the DAT during pregnancy.

† Women with single pregnancy and without prior caesarean section.

‡ Prelabour rupture of membranes, gestational hypertension, chronic hypertension, pre-eclampsia, eclampsia, cardiac/renal disease, chronic respiratory conditions, suspected fetal growth impairment, diabetes, vaginal bleeding during second half of pregnancy, condyloma acuminatum, confirmed HIV/AIDS, cholestasis and other obstetric or medical conditions.

DAT, decision analysis tool.

### Outcomes

**Figure 1:**
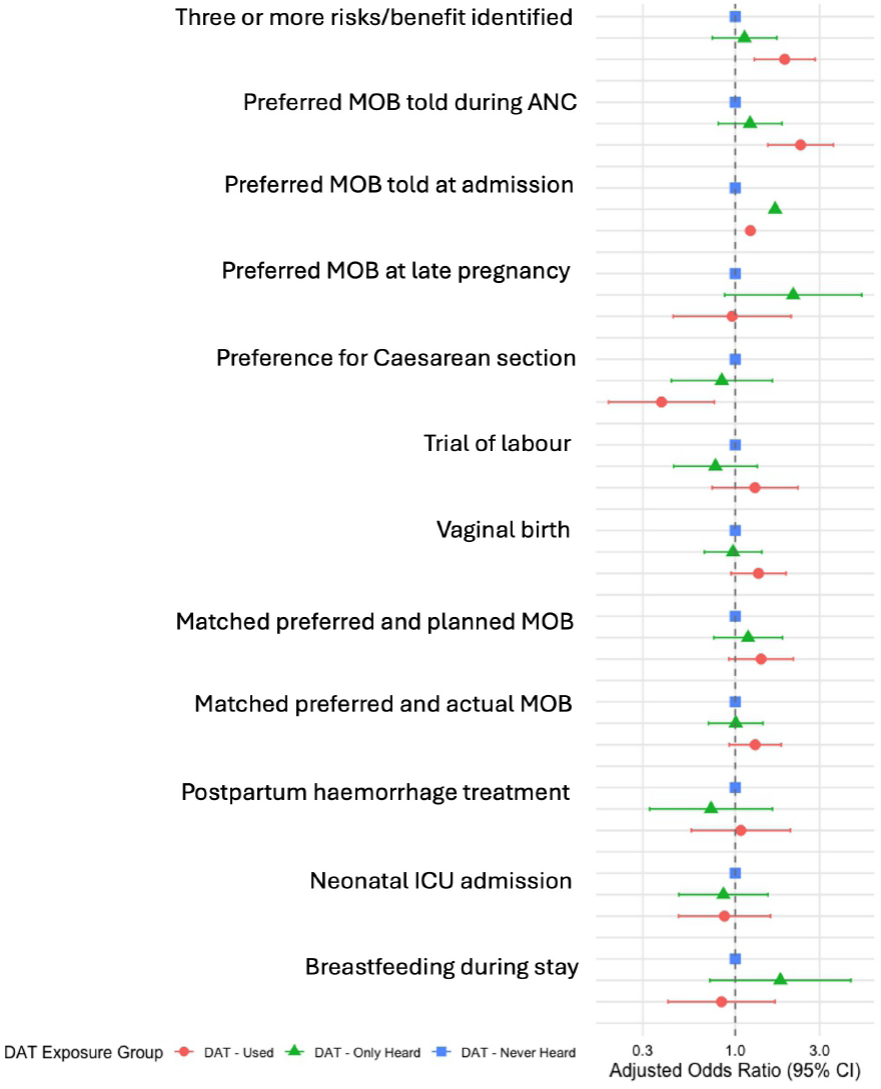
Associations between exposure and binary variable outcomes: adjusted ORs using logistic mixed models. ANC, antenatal care; DAT, decision analysis tool; ICU, intensive care unit; MOB, mode of birth.

Adjusted associations are presented numerically in table 3 and graphically in figures1  2 for binary and continuous outcomes, respectively. Bivariate analysis results are provided in online supplemental table S3.

**Table 3:** Associations between exposures and selected outcomes: multivariate analyses

Categorical variable outcome	Never heard N=1907 Reference N (%)	Only heard N=212 N (%)	Multivariate analysis Adjusted OR* (95% CI)	P value	Used N=249 N (%)	Multivariate analysis Adjusted OR* (95% CI)	P value
Three or more risks/benefits identified	359 (18.8)	44 (20.8)	1.1 (0.7 to 1.7)	0.578	61 (24.5)	1.9 (1.3 to 2.8)	0.001
Preferred MOB told during ANC	815 (47.4)	111 (57.2)	1.2 (0.8 to 1.8)	0.365	169 (76.8)	2.3 (1.5 to 3.6)	<0.001
Preferred MOB told at admission	364 (19.2)	91 (43.1)	1.7 (1.7 to 1.7)	<0.001	84 (33.7)	1.2 (1.2 to 1.2)	<0.001
Preferred MOB at late pregnancy	1552 (84.1)	192 (93.7)	2.1 (0.9 to 5.2)	0.098	224 (92.2)	1 (0.4 to 2.1)	0.915
Preference for CS	166 (9)	16 (7.8)	0.8 (0.4 to 1.6)	0.6	16 (6.6)	0.4 (0.2 to 0.8)	0.006
Trial of labour	1727 (91)	191 (90.5)	0.8 (0.4 to 1.3)	0.352	229 (92)	1.3 (0.7 to 2.3)	0.37
Vaginal birth	1282 (69.8)	142 (69.6)	1 (0.7 to 1.4)	0.878	169 (69.5)	1.4 (0.9 to 1.9)	0.098
Matched preferred and planned MOB	1380 (75.1)	170 (83.3)	1.2 (0.8 to 1.8)	0.465	204 (84)	1.4 (0.9 to 2.1)	0.118
Matched preferred and actual MOB	1174 (61.9)	142 (67.3)	1 (0.7 to 1.4)	0.973	172 (69.1)	1.3 (0.9 to 1.8)	0.134
PPH treatment	99 (5.2)	8 (3.8)	0.7 (0.3 to 1.6)	0.439	14 (5.6)	1.1 (0.6 to 2.1)	0.827
Neonatal ICU admission	261 (14.7)	17 (8.5)	0.9 (0.5 to 1.5)	0.601	16 (7.1)	0.9 (0.5 to 1.6)	0.645
Breastfeeding during stay	1822 (96)	205 (97.2)	1.8 (0.7 to 4.5)	0.21	236 (94.8)	0.8 (0.4 to 1.7)	0.614

Continuous variable outcome	Never Heard N=1907 Reference	Only heard N=212	Multivariate analysis Adjusted Beta† (95% CI)	P value	Used N=249	Multivariate analysis Adjusted Beta† (95% CI)	P value
Overall BES score, Median (IQR)	82 (75 to 95)	88 (75 to 98)	0.8 (−0.6 to 2.2)	0.259	95 (83 to 100)	1.9 (0.5 to 3.3)	0.006
Emotional support	83.33 (75 to 100)	83.33 (75 to 100)	1.5 (−0.6 to 3.6)	0.153	91.67 (75 to 100)	1.5 (−0.5 to 3.5)	0.131
Respect, dignity	93.75 (75 to 100)	93.75 (75 to 100)	0 (−1.6 to 1.6)	0.965	100 (88 to 100)	1.5 (0 to 3.1)	0.052
Patient-provider communication	75 (66.67 to 100)	83.33 (75 to 100)	1.2 (−0.7 to 3.1)	0.203	100 (75 to 100)	2.7 (0.8 to 4.5)	0.004

Data are presented as the median (IQR) for continuous variable outcomes, and as the number of women (%) for binary outcomes.

* OR of the multivariate mixed-effect logistic regression modelling to adjust for covariates with a random intercept model to take variability between hospitals into account.

† Coefficient of the multivariate mixed-effect linear regression modelling to adjust for covariates with a random intercept model to take variability between hospitals into account.

ANC, antenatal care; BES, birth experience and satisfaction; CS, caesarean section; ICU, intensive care unit; MOB, mode of birth; PPH, postpartum haemorrhage.

**Figure 2:**
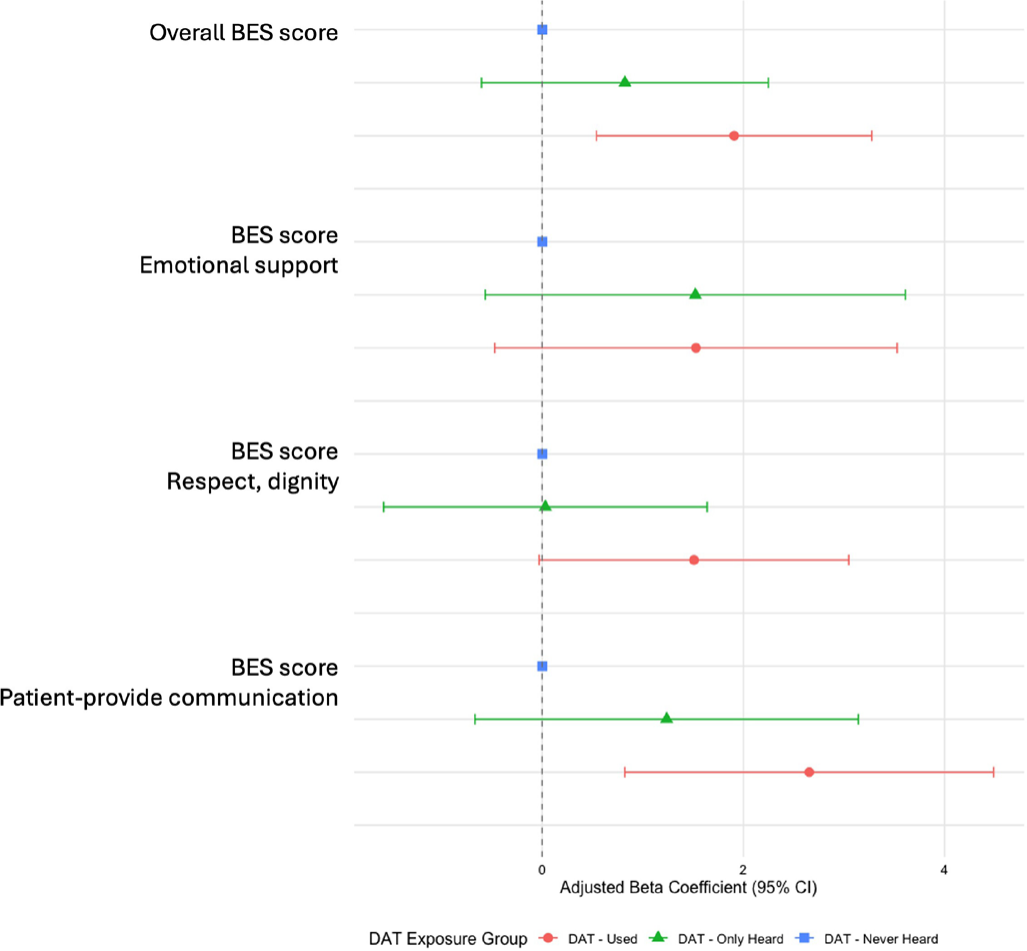
Associations between exposure and continuous variable outcomes: adjusted coefficients of linear mixed models. BES, birth experience and satisfaction; DAT, decision analysis tool.

Compared with women who had never heard about the DAT, those who used the tool were significantly more likely to identify three or more evidence-based risks and benefits for each MOB (adjusted OR, aOR 1.9; 95% CI 1.3 to 2.8; p=0.001), to express their preferred MOB during antenatal care (aOR 2.3; 95% CI 1.5 to 3.6; p<0.001) and at admission (aOR 1.2; 95% CI 1.2 to 1.2; p<0.001). DAT use was also associated with lower odds of preferring a CS in late pregnancy (aOR 0.4; 95% CI 0.2 to 0.8; p=0.006).

Regarding birth experience, adjusted linear regression models showed that overall BES scores were significantly higher among DAT users compared with the Never-heard group (β=1.9; 95% CI 0.5 to 3.3; p=0.006). The largest improvement was observed in the communication dimension (β=2.7; 95% CI 0.8 to 4.5; p=0.004).

Multilevel modelling showed that overall satisfaction scores were significantly influenced by the hospital where women gave birth, with intraclass correlation coefficients equal to 0.32. The between-hospital variance was 35.96% for BES scores indicating substantial facility-level effects on women’s experiences and perceptions of care. Full adjusted multivariate models are provided in online supplemental figure S2–S17.

Excluding women from Burkina Faso did not change the overall trends observed across outcome dimensions.

## Discussion

This multicountry, cross-sectional study revealed that among low-risk pregnant women, the use of the DAT was significantly associated with greater awareness of the risks and benefits of each MOB, with increased discussion with HCPs about women’s preference, a greater preference for vaginal birth, a more positive birth experience and greater satisfaction with care. The findings are consistent with what women said about the DAT. Most of them agreed that it was easy to use and said that it helped them talk to HCPs and clarify their birth preference. However, no significant differences were observed in other outcomes such as trial of labour, actual MOB or maternal and newborn health, among the exposure groups. Significant behavioural changes would have been needed from HCPs, in response to women’s use of the DAT, to have resulted in changes to birth outcomes. In contexts where caesareans occur without clear medical indication, ensuring that women can articulate their preferences, particularly their preference for vaginal birth, supports better alignment with respected maternal care.^19 25 26^ Facilitating informed discussions is consistent with women-centred care and does not conflict with clinicians’ responsibility to recommend vaginal birth when medically safest.^27^ In line with expert recommendations to reduce unnecessary CSs,^28 29^ our findings reinforce the need for multidimensional strategies that address both clinical and psychosocial determinants of MOB. Evidence from a recent scoping review indicates that women’s requests for planned caesarean without clinical indication are primarily motivated by fear of labour and a desire for control over the birth experience.^30^ Similar patterns emerged in our study settings, where women reported fear of labour pain, often exacerbated by limited access to epidural analgesia, and a wish to schedule the birth at an auspicious time.^19 31^ However, preferences for CS accounted for only a small proportion of caesarean births,^14^ suggesting that addressing women’s requests alone is insufficient. While it was not feasible within the QUALI-DEC trial to co-design additional interventions targeting women’s fear and difficult access to epidural, the DAT may help providers identify the reason for CS preference, explore fears and offer reassurance or mitigation strategies, as recommended by WHO guidance on respectful maternity care.^9^ The QUALI-DEC theory of change shows that birth outcomes and impact on CS use are linked to the implementation of all four components of the intervention.^18^ A scoping review of the effects of decision aids around childbirth supported the idea that these tools combined with other interventions seem to be important factors in improving care behaviour and health outcomes.^10^ The results of the QUALI-DEC trial’s process and effectiveness evaluation will reveal whether the multifaceted intervention produced the intended long-term outcomes. Importantly, our findings show that the use of the DAT was not associated with any harm to maternal or neonatal outcomes, which is consistent with evidence from studies conducted in high-income countries.^10^

Our study has several strengths. This study is the first to evaluate the impact of the DAT on the decision-making process for choosing the MOB for low-risk pregnant women in LMICs. Second, we address all four main objectives of the decision aids to perform a comprehensive evaluation of SDM regarding MOB in this context. While the study was observational and hospital-based, we controlled for confounding biases and cluster effect using appropriate statistical methods.

However, several limitations should be considered when interpreting these findings. First, the cross-sectional design limits causal inference. However, by adjusting for potential confounders, the analysis provides a more accurate estimate of the associations observed. Second, the number of women who used the DAT was relatively small compared with those who did not hear about it, limiting the subgroup analysis per country. Third, the level of DAT implementation varied across facilities. This could have diluted the observed associations, especially for care behaviour-related outcomes that require strong provider involvement. Fourth, women with major maternal health complications or perinatal death were excluded from the survey, preventing us from assessing the association of the DAT with severe morbidity. Fifth, other relevant outcomes such as women’s values about MOB, decisional conflict, decisional satisfaction and decisional regret were not measured. Sixth, the DAT was only available in formats that required women to read, which may have limited accessibility for women with low literacy. Seventh, the question of whether women told their care provider their preferred MOB reflects one-way communication of preference and does not necessarily capture whether a two-way discussion or SDM occurred. Finally, as data on women’s knowledge and preferences were collected after childbirth, recall and social desirability biases cannot be ruled out.

To maximise decision aid impacts for pregnant women, decision aids should be made available in multiple formats to accommodate different literacy levels, and efforts should be made to ensure a wide outreach. Future research should focus on why women do not use the DAT. Ideally, support from policymakers should be provided to ensure sustainability and broader uptake. When used effectively (not only heard about it), the DAT can help women prepare for SDM with their HCPs. At the same time, providers must have an honest and unbiased discussion with them and create space for women to express their values and preferences. This joint effort is essential to ensure that women in LMICs receive the care that is aligned with their preferences.

## Conclusions

Among low-risk pregnant women in Argentina, Burkina Faso, Thailand and Viet Nam, the use of the DAT was associated with significant differences in knowledge, value-based communication with HCPs, greater preference for vaginal birth and improved childbirth experience and satisfaction. These findings suggest that the DAT may have helped foster more respectful and women-centred care. Future research will assess the effects of the combination of the DAT with other interventions targeting HCPs on medical practice and perinatal outcomes.

## Supplementary material

10.1136/bmjgh-2025-022365 online supplemental file 1

10.1136/bmjgh-2025-022365 online supplemental appendix 1

## Data Availability

Data are available in a public, open access repository.
