# Supplementary material for: Implementation of a decision aid to promote shared decision-making on mode of birth in low-risk pregnant women: a cross-sectional study within the QUALI-DEC hybrid trial
Source: BMJ Glob Health. 2026 Mar 3;11(3):e022365. doi: 10.1136/bmjgh-2025-022365 (PMC12958879; doi:10.1136/bmjgh-2025-022365)
Supplement: online supplemental file 1 [file bmjgh-11-3-s001.pdf]

BMJ Global Health Author Reflexivity Statement

Adapted from Morton, B., Vercueil, A., Masekela, R., Heinz, E., Reimer, L., Saleh, S., Kalinga, C., Seekles, M., Biccard, B., Chakaya, J., Abimbola, S., Obasi, A. and Oriyo, N. (2022), Consensus statement on measures to promote equitable authorship in the publication of research from international partnerships. Anaesthesia, 77: 264-276. https://doi.org/10.1111/anae.15597

**Study conceptualisation**	
1. How does this study address local research and policy priorities?	This study addresses a major policy and research priority in low- and middle-income countries (LMICs): the overuse of caesarean section and the need to improve respectful, woman-centred maternity care. By evaluating the efficiency of the decision analysis tool (DAT) for mode of birth, the study directly aligns with national and global priorities to strengthen shared decision-making, reduce unnecessary interventions, and improve quality of care during childbirth. The DAT was developed and implemented as part of the QUALI-DEC project, which was explicitly designed to respond to country-specific contexts and policy needs in Argentina, Burkina Faso, Thailand, and Viet Nam.
2. How were local researchers involved in study design?	Local researchers from all four participating countries were actively involved in the study design through the QUALI-DEC consortium. They contributed to formative research, contextual adaptation of the DAT, selection of variables, and development of data collection tools. Their contextual knowledge ensured that the intervention and evaluation were culturally appropriate, feasible within local health systems, and responsive to local clinical practices and decision-making norms.
**Research management**	
3. How has funding been used to support the local research team(s)?	Project funding supported local research teams through dedicated personnel for coordination, data collection, supervision, and analysis at participating hospitals. Funds were also used to support training activities, stakeholder engagement, data management infrastructure, and participation of LMIC researchers in analysis workshops, consortium meetings, and dissemination activities.
**Data acquisition and analysis**	
4. How are research staff who conducted data collection acknowledged?	Research staff involved in data collection, including hospital coordinators, interviewers, and data managers are acknowledged through authorship where appropriate, acknowledgements in publications, and recognition in project reports. Their contributions were essential for implementing surveys, interviews, and routine data collection across study sites.
5. How have members of the research partnership been provided with access to study data?	All partners had access to study data according to their role through secure data-sharing platforms, including REDCap for quantitative data and ShareDocs for qualitative materials. Data access followed strict governance rules defined in the Consortium Agreement, ensuring equitable access while protecting participant confidentiality.

6. How were data used to develop analytical skills within the partnership?	Data analysis was conducted collaboratively under the supervision of the principal coordinator of the QUALI-DEC project. Researchers from the four participating countries were actively involved in the analytical process, with results discussed and interpreted jointly with the four country principal investigators. This collaborative approach facilitated shared learning, strengthened analytical skills within the partnership, and promoted cross-country comparison and interpretation of findings.
**Data interpretation**	
7. How have research partners collaborated in interpreting study data?	Interpretation of findings was conducted collaboratively through regular consortium meetings, joint analysis sessions, and iterative feedback on drafts. LMIC and European partners jointly discussed results, contextualised findings within country-specific health systems, and integrated qualitative and quantitative evidence to ensure balanced and locally grounded interpretations.
**Drafting and revising for intellectual content**	
8. How were research partners supported to develop writing skills?	LMIC researchers were actively involved in drafting manuscripts, revising content, and responding to reviewer comments. Senior researchers provided mentorship through iterative feedback, joint writing sessions, and support during the publication process, contributing to capacity building in scientific writing.
9. How will research products be shared to address local needs?	Research outputs will be disseminated through international peer-reviewed publications in English and shared locally through national and subnational dissemination workshops. Findings will be presented to policymakers, health facility managers, and healthcare providers to support local decision-making, inform practice, and guide future implementation of decision-support tools in maternity care.
**Authorship**	
10. How is the leadership, contribution and ownership of this work by LMIC researchers recognised within the authorship?	LMIC researchers are recognised through leading and senior authorship positions, reflecting their substantive contributions to study design, data collection, analysis, and interpretation. Authorship decisions follow transparent consortium principles that value intellectual leadership and local ownership.
11. How have early career researchers across the partnership been included within the authorship team?	Early career researchers were included as co-authors and actively engaged in data analysis, interpretation, and manuscript drafting. The project provided opportunities for mentorship, skill development, and visibility within international publications.
12. How has gender balance been addressed within the authorship?	The authorship team reflects gender balance, with substantial representation of women researchers across senior, mid-career, and early career positions, consistent with the gender composition of the maternal health research field and the QUALI-DEC consortium.

**Training**	
13. How has the project contributed to training of LMIC researchers?	The project contributed to training through hands-on involvement in research implementation, and participation in international scientific exchanges. These activities strengthened research, analytical, and dissemination skills among LMIC partners.
**Infrastructure**	
14. How has the project contributed to improvements in local infrastructure?	The project strengthened local research infrastructure through the introduction of secure digital data systems (e.g. REDCap), improved data management processes, and capacity for routine monitoring and evaluation within participating hospitals. It also supported institutional capacity for quality improvement in maternity care.
**Governance**	
15. What safeguarding procedures were used to protect local study participants and researchers?	The study adhered to strict ethical and governance procedures, including ethical approvals from national and institutional review boards in all participating countries. Informed consent was obtained from all participants, data were anonymised or pseudonymised, and secure data storage systems were used in compliance with GDPR principles. Safeguards were in place to ensure voluntary participation, confidentiality, and protection of both participants and research staff.
