# Supplementary material for: Implementation of a decision aid to promote shared decision-making on mode of birth in low-risk pregnant women: a cross-sectional study within the QUALI-DEC hybrid trial
Source: BMJ Glob Health. 2026 Mar 3;11(3):e022365. doi: 10.1136/bmjgh-2025-022365 (PMC12958879; doi:10.1136/bmjgh-2025-022365)
Supplement: online supplemental appendix 1 [file bmjgh-11-3-s002.pdf]

APPENDIX

Supplementary information

Text

Text S1: Inclusion of participants and data collection

Table

Table S1: Definition of decision aid outcomes by objective

Table S2: Evidence-based risks and benefits of each mode of delivery with which a woman should be familiar to make an informed choice

Table S3: Associations between exposures and selected outcomes: bivariate analyses.

Figure

Figure S1: Flow chart representing the recruitment of participants into the second (post intervention) cross-sectional survey of Quali-Dec trial

Figure S2: Multilevel linear regression model for the association of risks/benefit identified with exposure, adjusted on selected confounders

Figure S3: Multilevel logistic regression model for the association of preferred mode of birth told during ANC with exposure, adjusted on selected confounders

Figure S4: Multilevel logistic regression model for the association of preferred mode of birth told at admission with exposure, adjusted on selected confounders

Figure S5: Multilevel logistic regression model for the association of women with preferred mode of birth at late pregnancy with exposure, adjusted on selected confounders

Figure S6: Multilevel logistic regression model for the association of preference for caesarean section with exposure, adjusted on selected confounders

Figure S7: Multilevel logistic regression model for the association of trial of labour with exposure, adjusted on selected confounders

Figure S8: Multilevel logistic regression model for the association of vaginal birth with exposure, adjusted on selected confounders

Figure S9: Multilevel logistic regression model for the association of matched preference and planned mode of birth with exposure, adjusted on selected confounders

Figure S10: Multilevel logistic regression model for the association of matched preference and actual mode of birth with exposure, adjusted on selected confounders

Figure S11: Multilevel logistic regression model for the association of women with postpartum haemorrhage treatment with exposure, adjusted on selected confounders

Figure S12: Multilevel logistic regression model for the association of neonatal ICU admission with exposure, adjusted on selected confounders

Figure S13: Multilevel logistic regression model for the association of breastfeeding during stay with exposure, adjusted on selected confounders

Figure S14: Multilevel linear regression model for the association of BES-emotional support score with exposure, adjusted on selected confounders

Figure S15: Multilevel linear regression model for the association of BES-respect and dignity score with exposure, adjusted on selected confounders

Figure S16: Multilevel linear regression model for the association of BES-communication score with exposure, adjusted on selected confounders

Figure S17: Multilevel linear regression model for the association of overall birth experience and satisfaction score with exposure, adjusted on selected confounders

Text S1: QUALI-DEC cross-sectional survey

Prior to the cross-sectional survey, a baseline formative research was first conducted in 2020 as part of the QUALI-DEC project. This formative research collected the relevant institutional and organisational data in each hospital to adapt the intervention to the context. This data was collected by each local principal investigator using a data collection form. Data were then extracted by the principal data manager and the final database was controlled by each country principal investigator.

The design of both cross-sectional surveys was based on the World Health Organization (WHO) Global Health Survey of Maternal and Perinatal Health[28]. In each country, data collection took place daily in all hospitals until the required number of participants was reached, but still continuing for two full weeks if the sample size was reached earlier. To achieve this goal, we estimated that 5–6 post-partum women had to be interviewed each day. For hospitals with a high volume of activity, a random sampling of women was used to reach 10 selected women per day, among those who had given birth the previous day. We assumed that 4–5 women would refuse to participate or would be ineligible, which would leave us with 5–6 recruitments and interviews per day. As for the first cross-sectional survey, the second survey period depended on the number of births in each hospital and ranged from 14 days to 118 days between October 16th 2023 and September 16th 2024.

A data collector assigned all randomly selected women an identification number and assessed their eligibility using a screening form. If the eligibility criteria were met, a social scientist approached the postpartum women and offered them to participate during their hospital stay. A consent form was completed when the women agreed to participate and the social scientist interviewed the participants face-to-face using a tablet for data collection form. The questionnaire was developed based on a literature review and validated by the experts comprising the QUALI-DEC project team. A pilot survey was conducted to test the

questionnaire, which was modified where necessary. The information collected was organized into seven modules: women's characteristics, antenatal care and preference for mode of birth; birth outcomes; women's knowledge about modes of birth; labour companionship; women's birth experience and satisfaction; gender dimensions and social equity; wealth characteristics and out-of-pocket expenses. Information on medical history, pregnancy, labour and delivery were simultaneously extracted for all included women by a clinical data collector from medical records using a standard data collection form. Data were entered in duplicate by two local data abstractors into an electronic system specifically designed with validation checks (REDCap®). Final consistency checks were carried out by the principal data manager, with regular support from the country-level data managers.

Table S1. Definition of decision aid outcome by objective (QUALI-DEC).

DA objective	Outcome	Information collected from interviewed women (or extracted from the medical record)	Definition of the outcome
Quality of decision	Risk/benefits identified	What would you say are the benefits of vaginal delivery and caesarean section? What would you say are the risks or inconveniences of vaginal birth and caesarean section?	Proportion of women identified 3 or more correct risks/benefits of vaginal birth and CS among the six listed in Table S2.
Quality of decision-making process	Preferred MOB told during ANC	Did you tell your healthcare provider about your preferred mode of birth?	Proportion of women who answered 'Yes – during antenatal care'
	Preferred MOB told at admission	Did you tell your healthcare provider about your preferred mode of birth?	Proportion of women who answered 'Yes – when I arrived at the hospital for birth'
Care behaviour	Preferred MOB at late pregnancy	Towards the end of your pregnancy, did you have a preferred mode of birth? For example, vaginal birth or caesarean section?	Proportion of women who answered 'Yes'
	Preference for CS	What was your preferred mode of birth towards the end of pregnancy?	Proportion of women who answered 'Caesarean section'
	Trial of labour	Which was the onset of labour? (information extracted from the medical record)	Proportion of women with spontaneous or induced labour
	Vaginal birth	Which was the final mode of birth (information extracted from the medical record)	Proportion of women with vaginal birth (spontaneous or assisted by forceps/vacuum)
	Matched preferred and planned MOB	What was your preferred mode of birth towards the end of pregnancy? AND	Proportion of women who had an onset of labour according to her preferred mode of birth

		Which was the onset of labour? (information extracted from the medical record)	
	Matched preferred and actual MOB	What was your preferred mode of birth towards the end of pregnancy?<br>AND<br>Which was the final mode of birth (information extracted from the medical record)	Proportion of women who had a final mode of birth according to her preferred mode of birth
Health outcomes	PPH treatment	Following information extracted from the medical record:<br>- Was any uterotonic given?<br>- Did the patient receive a blood transfusion?<br>- For what indication?<br>- Was any interventions used?	Proportion of women who had uterotonic, blood transfusion, hysterectomy, laparotomy or any other intervention for postpartum haemorrhage treatment
	Neonatal ICU admission	Admission of the newborn to ICU / Special care unit (information extracted from the medical record)	Proportion of women whose newborns were admitted to neonatal intensive care unit
	Breastfeeding	Following information collected from interviewed women:<br>- How long after birth did you first put your baby at your breast?<br>- What was given to your baby?<br>- Are you currently breastfeeding?	Women were classified as having given breast milk at any time during the hospital stay if they reported initiating breastfeeding (from the question on time to first breastfeed) or indicated that breast milk was given to the newborn. The question on current breastfeeding was used to verify response consistency.
	Satisfaction regarding emotional support	1. Labour can be a painful and difficult time for women. Do you agree with this statement: “I am satisfied with my ability to cope with pain during labour”?<br>2. You may have been looking forward to holding your baby after he/she was born, and sometimes this can take some time. Do you	The satisfaction score was calculated as the ratio between the woman’s observed score and the maximum possible score for the items applicable to her. Each item is scored from 1 to 4. Applicability of items varied by clinical context:

		agree with this statement: "I am satisfied with the amount of time that passed after the birth, before I first held my baby"?<br>3. You were probably looking forward to feeding your baby for the first time. Do you agree with this statement: "I am satisfied with the amount of time that passed before I first fed my baby "?	All three items (maximum score = 12): used for women who laboured and were not separated from their newborn.<br><br>Two items (items 2 and 3 only; maximum score = 8): used for women with a planned caesarean section before labour, for whom item 1 (coping with labour pain) was not applicable.<br><br>One item (item 1 only; maximum score = 4): used for women separated from their newborn after birth (e.g., NICU admission), for whom items 2 and 3 (holding and feeding the baby) were not applicable. Each item is scored 1 to 4 (1: strongly disagree; 2: disagree; 3: agree; 4: strongly agree)
	Satisfaction regarding respect and dignity	4.Do you agree with this statement: "I am satisfied with the amount of time the health workers spent with me during labour"?<br>5. Do you agree with this statement: "I am satisfied with the attitude of health workers during labour and birth"?<br>6. Sometimes the labour room can get busy. Do you agree with this statement: "I feel that my privacy was respected during examinations and treatments"?<br>7. Based your experience during labour and childbirth, would you agree or disagree with this statement: "Overall, I felt well supported during labour and childbirth".	Ratio between the observed score and the maximum score of the 4-items BES scale. Each item is scored 1 to 4 (1: strongly disagree; 2: disagree; 3: agree; 4: strongly agree)
	Satisfaction regarding patient-provider communication	8. Do you agree with this statement: "During my stay at the hospital for childbirth, health	Ratio between the observed score and the maximum score of the 3-items BES scale. Each item is scored 1

		workers informed me about decisions that they took regarding my care"?<br>9. You probably had some concerns or fears during your stay in the hospital for childbirth. Do you agree with this statement: "During my stay at the hospital for childbirth, I feel that I had the opportunity to discuss any fears or concerns I had with a health worker"?<br>10. You may have had some preferences about how you wanted to give birth. Do you agree with this statement: "I had the opportunity to discuss my preferences or requests with a health worker during your stay in the hospital for birth"?	to 4 (1: strongly disagree; 2: disagree; 3: agree; 4: strongly agree)
	Overall birth experience and satisfaction (BES) with intrapartum care	10 items above	The score was calculated as the ratio between the woman's observed score and the maximum possible score for the items applicable to her. Each item is scored from 1 to 4. Applicability varied by clinical circumstances:<br><br>All 10 items (maximum score = 40): used for women who laboured and were not separated from their newborn.<br><br>Nine items (maximum score = 36): used for women with a planned caesarean section before labour, for whom item 1 (coping with labour pain) was not applicable.<br><br>Eight items (maximum score = 32): used for women separated from their newborn after birth, for whom

	items 2 and 3 (holding and feeding the baby immediately after birth) were not applicable.<br><br>The final score therefore reflects each woman's experience relative to the set of items appropriate for her situation.. Each item is scored 1 to 4 (1: strongly disagree; 2: disagree; 3: agree; 4: strongly agree)

Table S2: Evidence-based risks and benefits of each mode of delivery with which a woman should be familiar to make an informed choice

	**Correct answers**	
**Risks or benefits**	**Advantages or disadvantages of planned vaginal birth**	**Advantages or disadvantages of planned caesarean section**
VB has shorted hospital stay and faster recovery	Shorter hospital stays, faster recovery	Longer hospital stays, slower recovery, postoperative pain
VB increases the likelihood of successful breastfeeding	Increases the chances of successful breastfeeding	Reduces breastfeeding success
CS carries surgery-related risks	Reduce surgery-related risks	Increased risk of serious complications
CS increases the risk of complications in future pregnancies	Reduce the risk of complications in future pregnancies	Increased risk of future pregnancy complications
VB carries the risk of requiring an emergency CS during labour	Increase the risk of emergency caesarean section during labour or delivery	Reduce the risk of emergency caesarean section in labour
CS increases the risk of respiratory complications in newborns		Higher risk of cardio-respiratory complications for your baby<br>OR<br>Increased risk of respiratory disorders after birth (optional: when caesarean is planned before 39 weeks)

Table S3. Associations between exposures and selected outcomes: bivariate analyses.

Categorial variable outcome	Never heard N=1907 Reference N (%)	Only heard N = 212 N (%)	Bivariate analysis Crude OR (95% CI)	P-value	Used N = 249 N (%)	Bivariate analysis Crude OR (95% CI)	P-value
Three or more risks/benefits identified	359 (18.8)	44 (20.8)	1.1 [0.7 ; 1.7]	0.587	61 (24.5)	1.9 [1.3 ; 2.8]	0.001
Preferred MOB told during ANC	815 (47.4)	111 (57.2)	1.3 [0.9 ; 1.9]	0.106	169 (76.8)	3 [2.1 ; 4.4]	<0.001
Preferred MOB told at admission	364 (19.2)	91 (43.1)	1.7 [1.2 ; 2.4]	0.006	84 (33.7)	1.3 [0.9 ; 1.9]	0.146
Preferred MOB at late pregnancy	1552 (84.1)	192 (93.7)	2.1 [1.1 ; 4]	0.025	224 (92.2)	1.4 [0.8 ; 2.5]	0.226
Preference for CS	166 (9)	16 (7.8)	0.8 [0.4 ; 1.3]	0.337	16 (6.6)	0.6 [0.3 ; 1]	0.052
Trial of labour	1727 (91)	191 (90.5)	1 [0.6 ; 1.6]	0.915	229 (92)	1.2 [0.7 ; 2]	0.459
Vaginal birth	1282 (69.8)	142 (69.6)	1 [0.7 ; 1.4]	0.93	169 (69.5)	1.1 [0.8 ; 1.5]	0.57
Matched preferred and planned MOB	1380 (75.1)	170 (83.3)	1.4 [0.9 ; 2.2]	0.097	204 (84)	1.4 [1.0 ; 2.2]	0.078
Matched preferred and actual MOB	1174 (61.9)	142 (67.3)	1.1 [0.8 ; 1.5]	0.684	172 (69.1)	1.2 [0.9 ; 1.7]	0.238
PPH treatment	99 (5.2)	8 (3.8)	0.6 [0.3 ; 1.4]	0.234	14 (5.6)	1 [0.5 ; 1.9]	0.984
Neonatal ICU admission	261 (14.7)	17 (8.5)	0.7 [0.4 ; 1.3]	0.263	16 (7.1)	0.6 [0.3 ; 1.1]	0.084
Breastfeeding during stay	1822 (96)	205 (97.2)	1.9 [1.9 ; 1.9]	<0.001	236 (94.8)	0.9 [0.9 ; 0.9]	<0.001
**Continuous variable outcome**	**Never Heard N=1907 Reference**	**Only heard N = 212**	**Bivariate analysis (Kruskal Wallis test)**	**P-value**	**Used N = 249**	**Bivariate analysis (Kruskal Wallis test)**	**P-value**
Overall BES score, Median (IQR)	82 [75 ; 95]	88 [75 ; 98]	8.9617	0.003	95 [83 ; 100]	57.694	<0.001
Emotional support	83.33 [75 ; 100]	83.33 [75 ; 100]	4.9007	0.027	91.67 [75 ; 100]	20.414	<0.001
Respect, dignity	93.75 [75 ; 100]	93.75 [75 ; 100]	0.4714	0.492	100 [88 ; 100]	40.00	<0.001
Patient-provide communication	75 [66.67 ; 100]	83.33 [75 ; 100]	35.507	<0.001	100 [75 ; 100]	112.59	<0.001

Data are presented as the median (interquartile range) for continuous variable outcomes, and as the number of women (%) for binary outcomes.
[a]Coefficient of the multivariate mixed-effect linear regression modelling to adjust for covariates with a random intercept model to take variability between hospitals into account;
[b]Odd ratio of the multivariate mixed-effect logistic regression modelling to adjust for covariates with a random intercept model to take variability between hospitals into account.
MOB: mode of birth; PPH: postpartum haemorrhage; ICU: intensive care unit; ANC: antenatal care

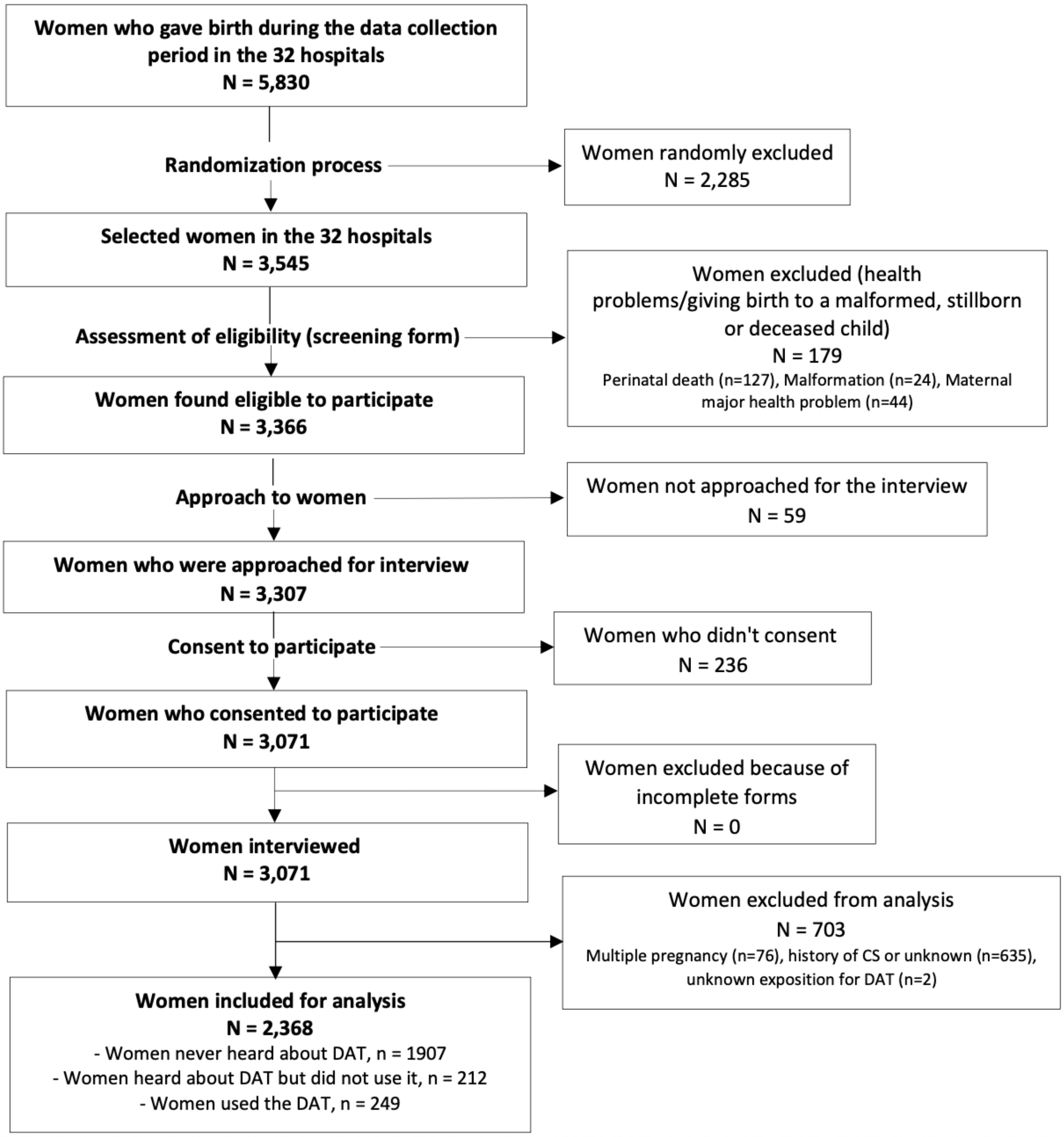

Figure S1. Flowchart representing the recruitment of participants into the cross-sectional survey.

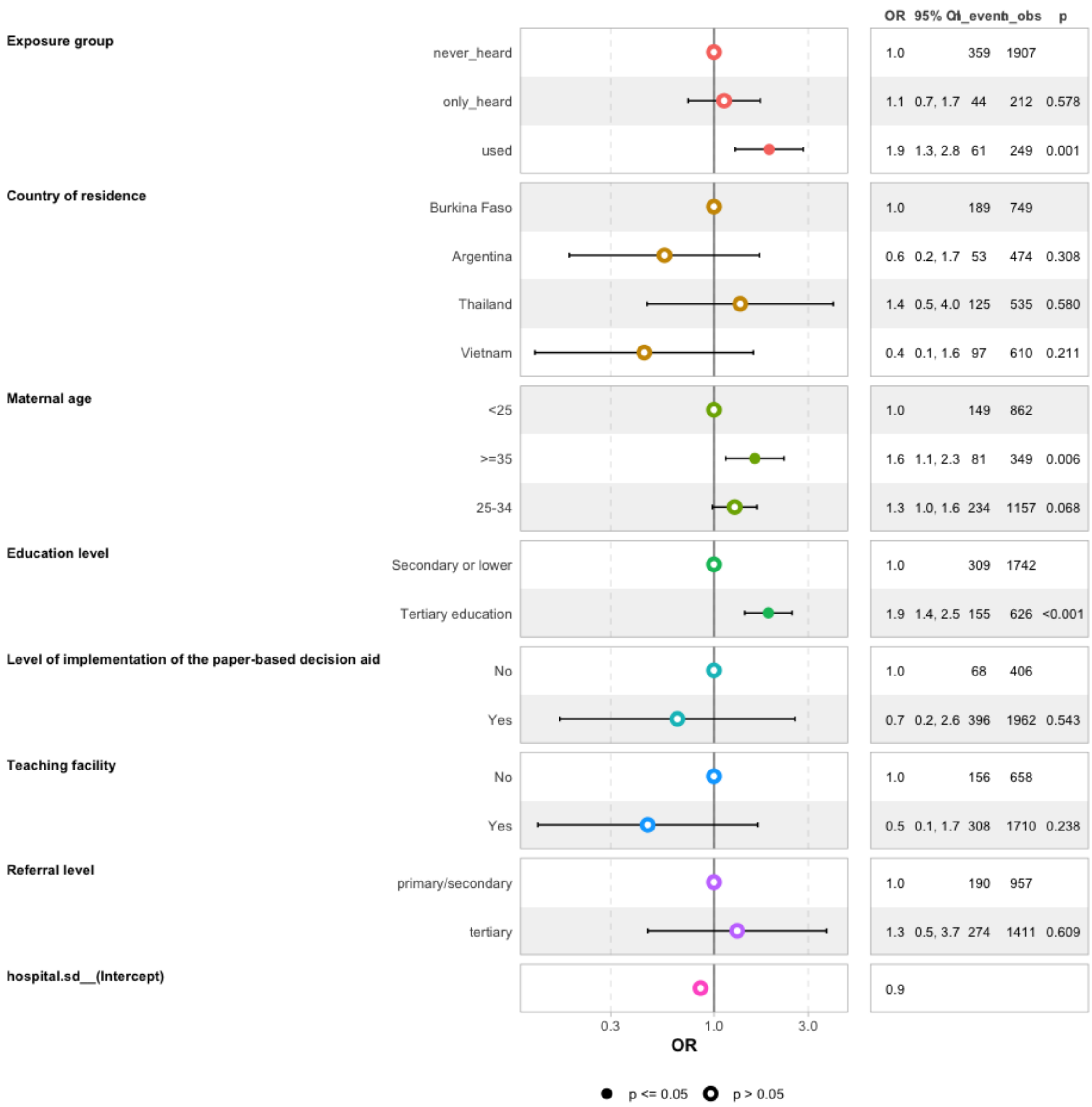

Figure S2: Multilevel linear regression model for the association of risks/benefit identified with exposure, adjusted on selected confounders

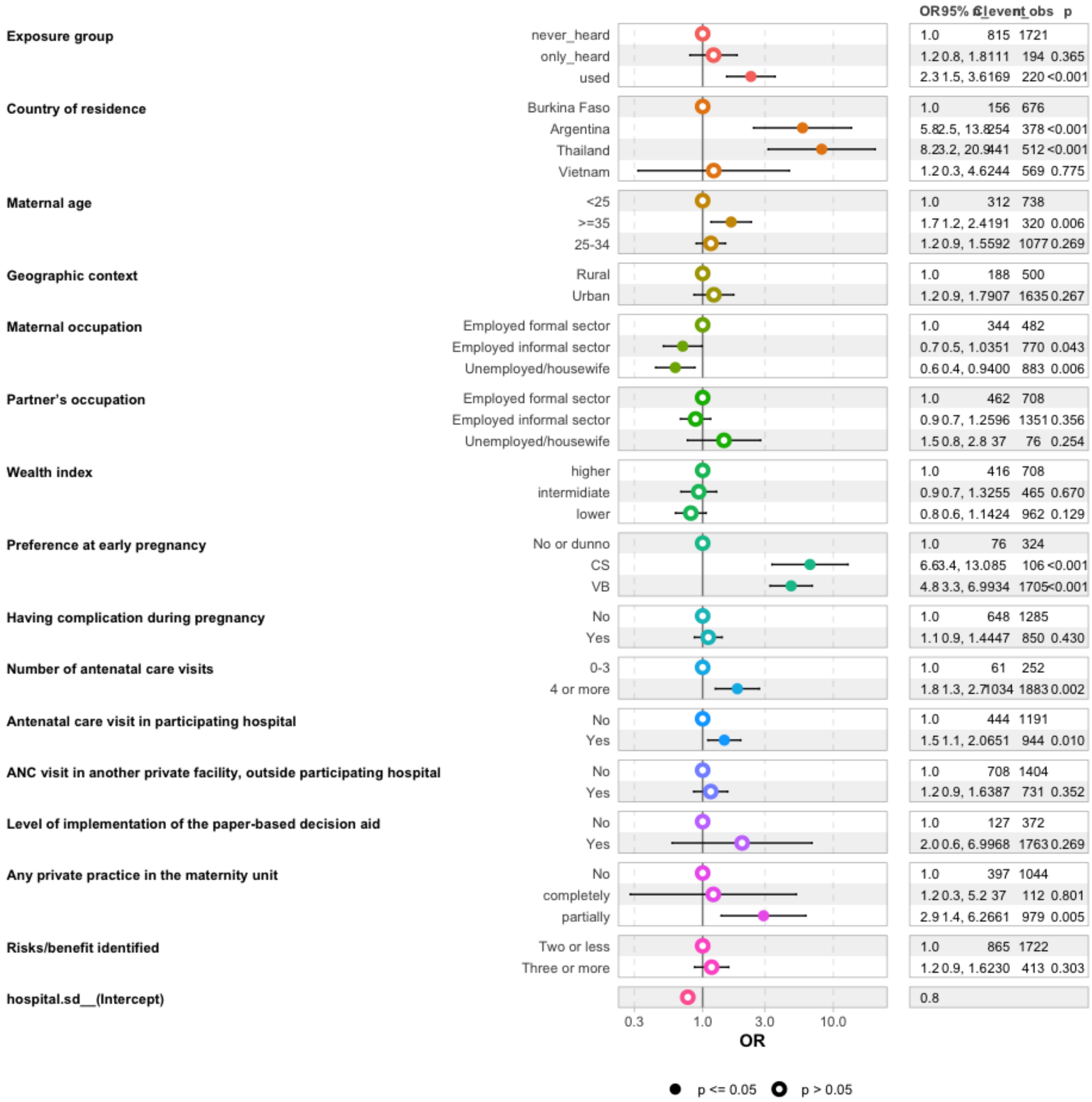

Figure S3: Multilevel logistic regression model for the association of preferred mode of birth told during ANC with exposure, adjusted on selected confounders

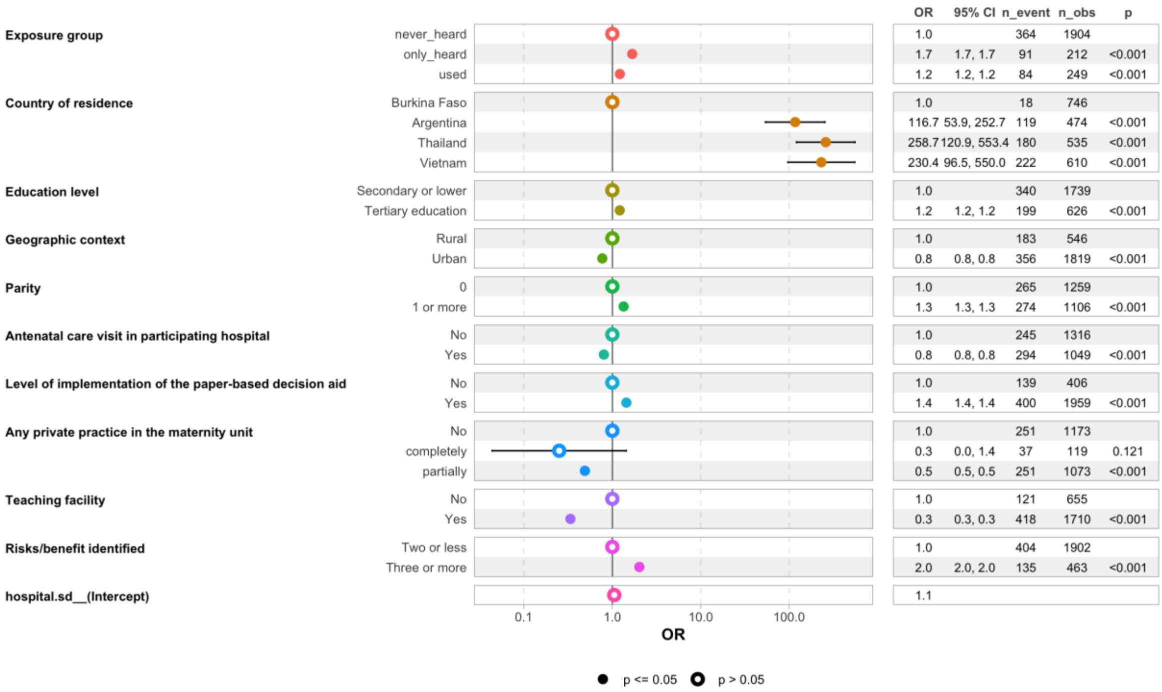

		OR	95% CI	n_event	n_obs	p
Exposure group	never_heard	1.0		364	1904	
	only_heard	1.7	1.7, 1.7	91	212	<0.001
	used	1.2	1.2, 1.2	84	249	<0.001
Country of residence	Burkina Faso	1.0		18	746	
	Argentina	116.7	53.9, 252.7	119	474	<0.001
	Thailand	258.7	120.9, 553.4	180	535	<0.001
	Vietnam	230.4	96.5, 550.0	222	610	<0.001
Education level	Secondary or lower	1.0		340	1739	
	Tertiary education	1.2	1.2, 1.2	199	626	<0.001
Geographic context	Rural	1.0		183	546	
	Urban	0.8	0.8, 0.8	356	1819	<0.001
Parity	0	1.0		265	1259	
	1 or more	1.3	1.3, 1.3	274	1106	<0.001
Antenatal care visit in participating hospital	No	1.0		245	1316	
	Yes	0.8	0.8, 0.8	294	1049	<0.001
Level of implementation of the paper-based decision aid	No	1.0		139	406	
	Yes	1.4	1.4, 1.4	400	1959	<0.001
Any private practice in the maternity unit	No	1.0		251	1173	
	completely	0.3	0.0, 1.4	37	119	0.121
	partially	0.5	0.5, 0.5	251	1073	<0.001
Teaching facility	No	1.0		121	655	
	Yes	0.3	0.3, 0.3	418	1710	<0.001
Risks/benefit identified	Two or less	1.0		404	1902	
	Three or more	2.0	2.0, 2.0	135	463	<0.001
hospital.sd__(Intercept)		1.1				

Figure S4: Multilevel logistic regression model for the association of preferred mode of birth told at admission with exposure, adjusted on selected confounders

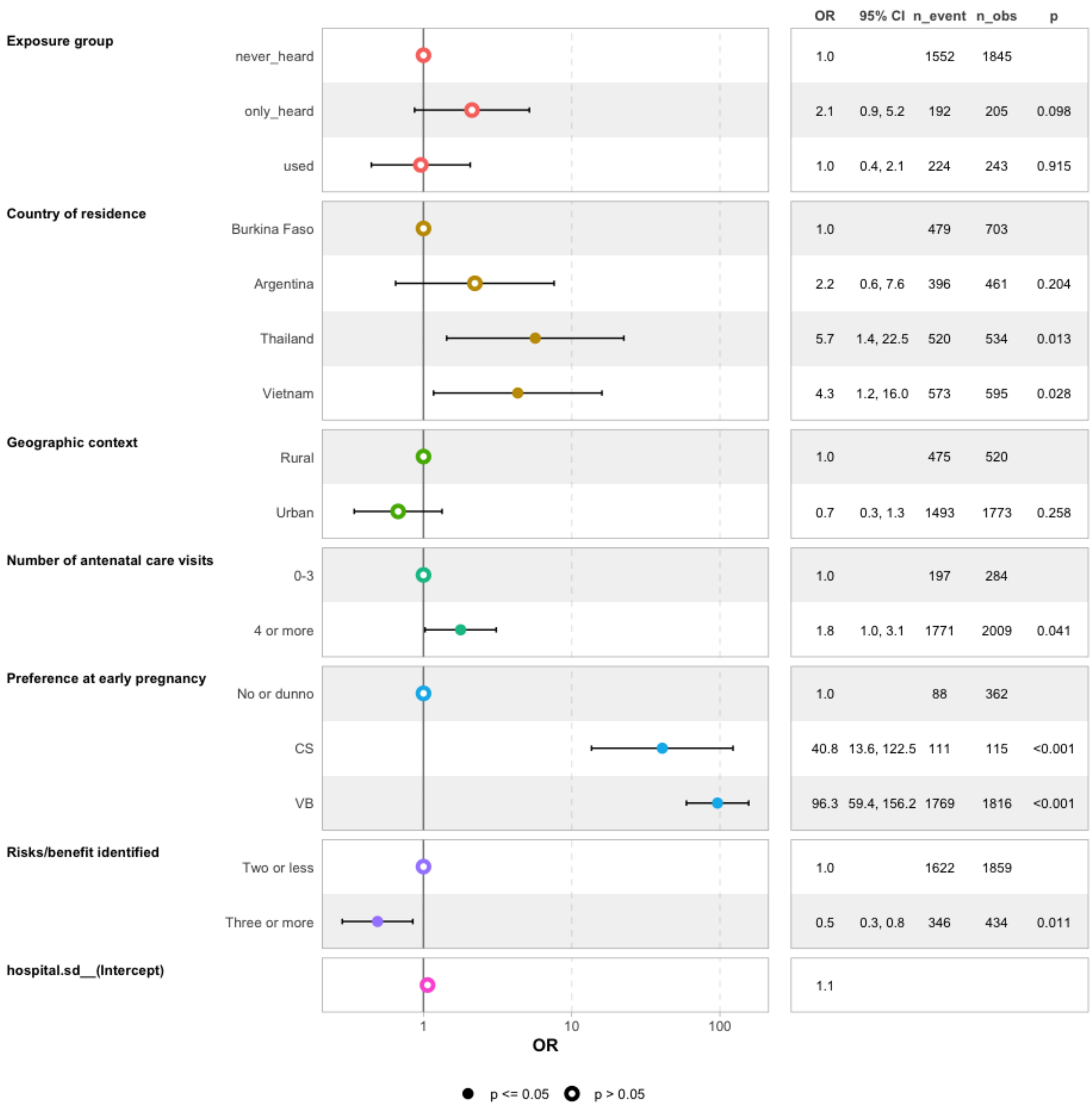

Figure S5: Multilevel logistic regression model for the association of women with preferred mode of birth at late pregnancy with exposure, adjusted on selected confounders

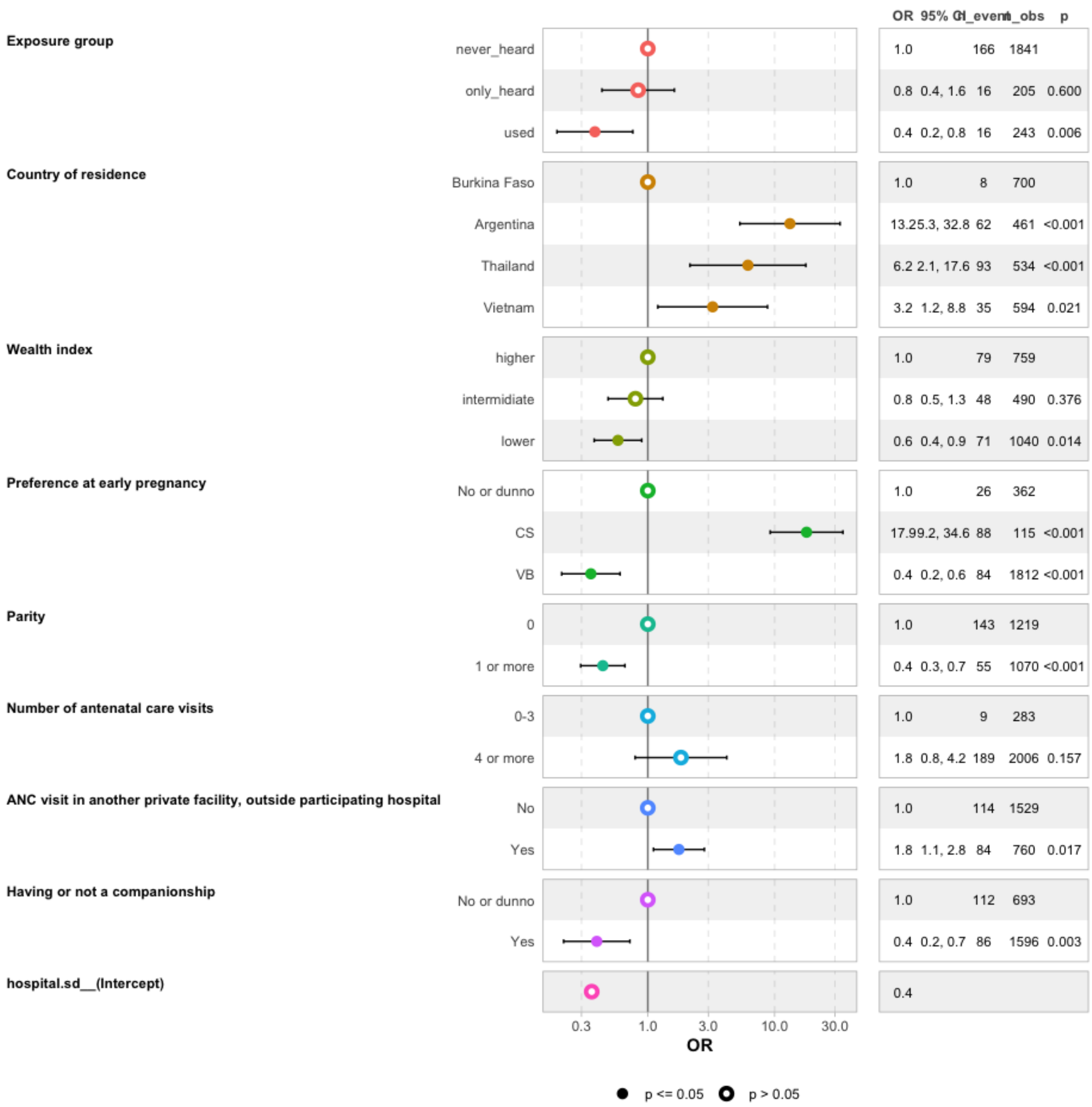

Figure S6: Multilevel logistic regression model for the association of preference for caesarean section with exposure, adjusted on selected confounders

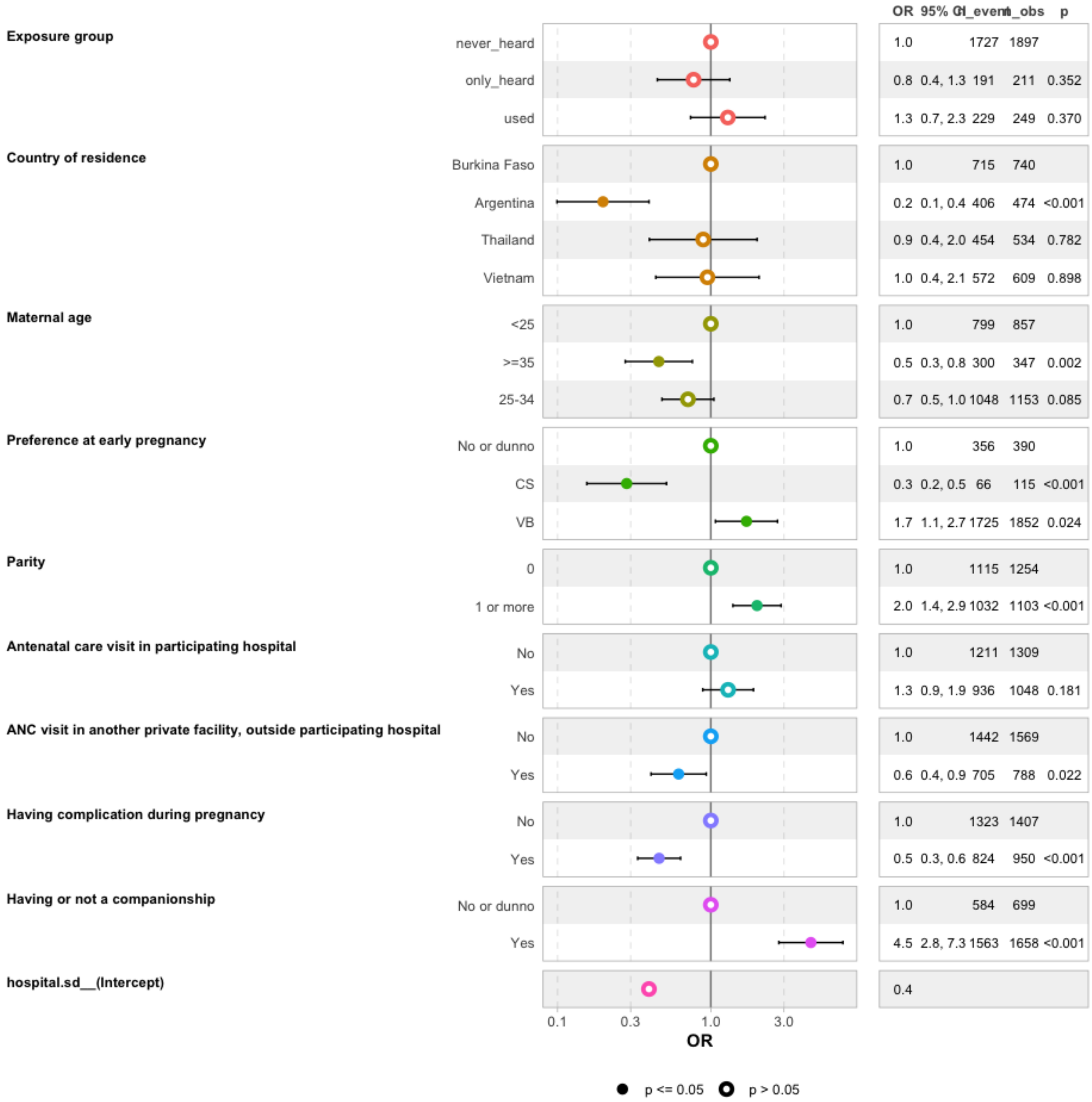

Figure S7: Multilevel logistic regression model for the association of trial of labour with exposure, adjusted on selected confounders

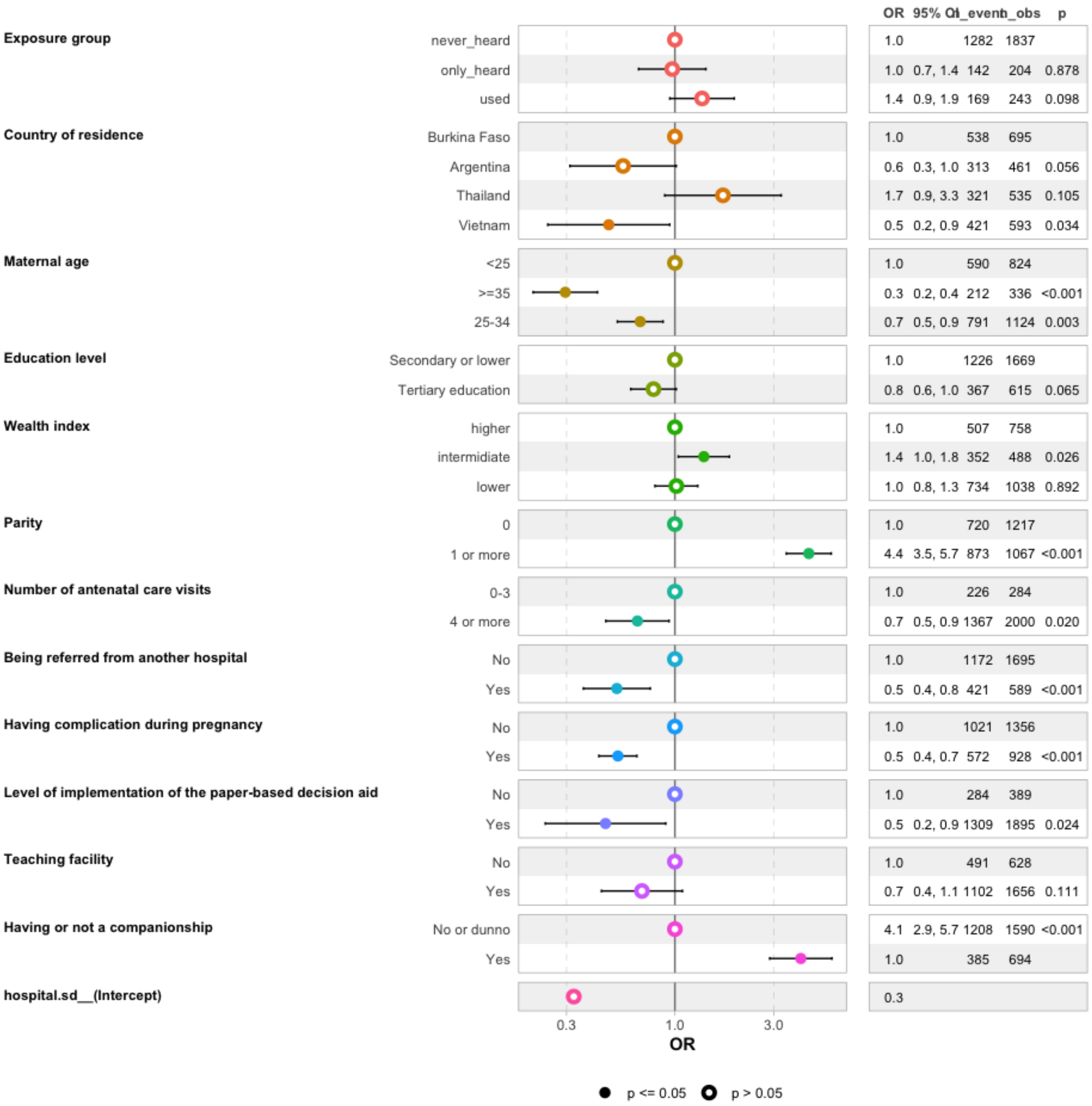

Figure S8: Multilevel logistic regression model for the association of vaginal birth with exposure, adjusted on selected confounders

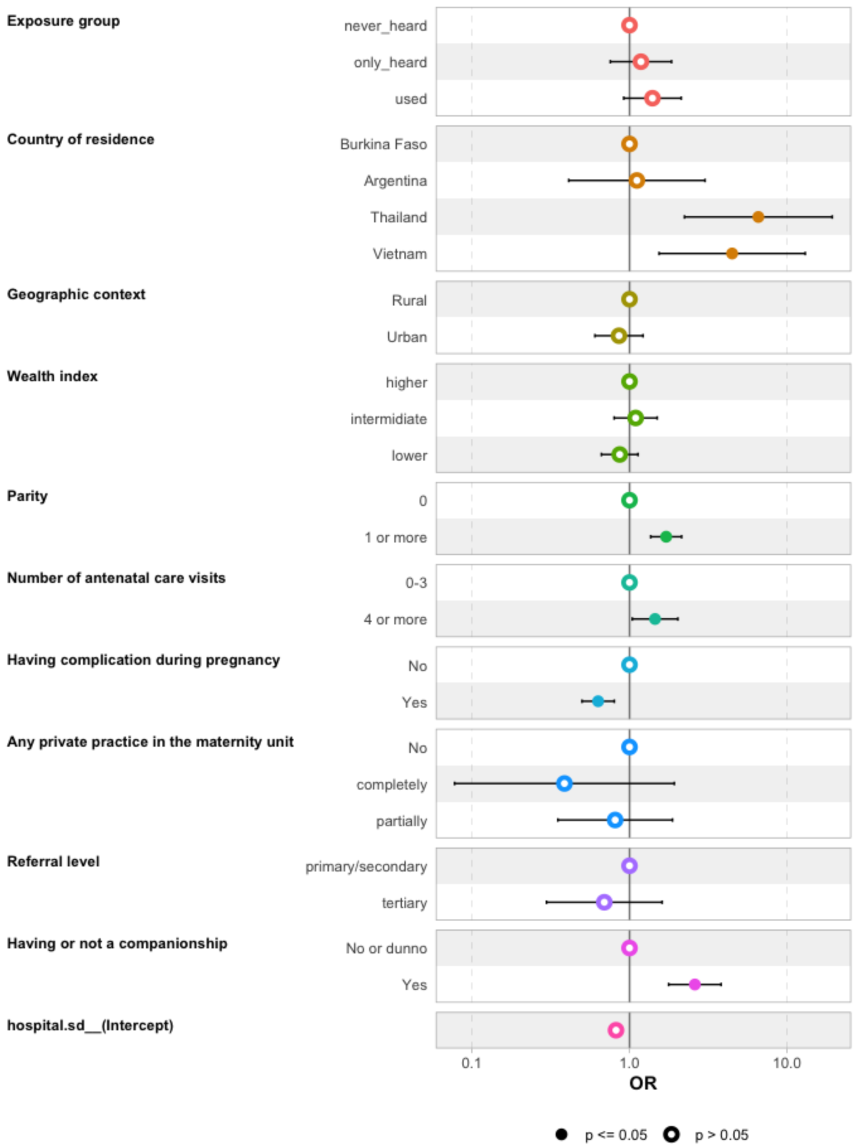

Variable	Level	OR	95% CI	n_event	n_obs	p
Exposure group	never_heard	1.0		1380	1837	
	only_heard	1.2	0.8, 1.8	170	204	0.465
	used	1.4	0.9, 2.1	204	243	0.118
Country of residence	Burkina Faso	1.0		455	695	
	Argentina	1.1	0.4, 3.0	330	461	0.832
	Thailand	6.6	2.2, 19.4	449	535	<0.001
	Vietnam	4.5	1.5, 13.0	520	593	0.006
Geographic context	Rural	1.0		448	519	
	Urban	0.9	0.6, 1.2	1306	1765	0.391
Wealth index	higher	1.0		582	758	
	intermidiate	1.1	0.8, 1.5	374	488	0.577
	lower	0.9	0.7, 1.1	798	1038	0.294
Parity	0	1.0		886	1217	
	1 or more	1.7	1.4, 2.1	868	1067	<0.001
Number of antenatal care visits	0-3	1.0		179	284	
	4 or more	1.5	1.0, 2.0	1575	2000	0.028
Having complication during pregnancy	No	1.0		1065	1356	
	Yes	0.6	0.5, 0.8	689	928	<0.001
Any private practice in the maternity unit	No	1.0		865	1139	
	completely	0.4	0.1, 1.9	96	116	0.246
	partially	0.8	0.4, 1.9	793	1029	0.623
Referral level	primary/secondary	1.0		717	920	
	tertiary	0.7	0.3, 1.6	1037	1364	0.393
Having or not a companionship	No or dunno	2.6	1.8, 3.8	1206	1590	<0.001
	Yes	1.0		548	694	
hospital.sd__(Intercept)		0.8				

Figure S9: Multilevel logistic regression model for the association of matched preference and planned mode of birth with exposure, adjusted on selected confounders

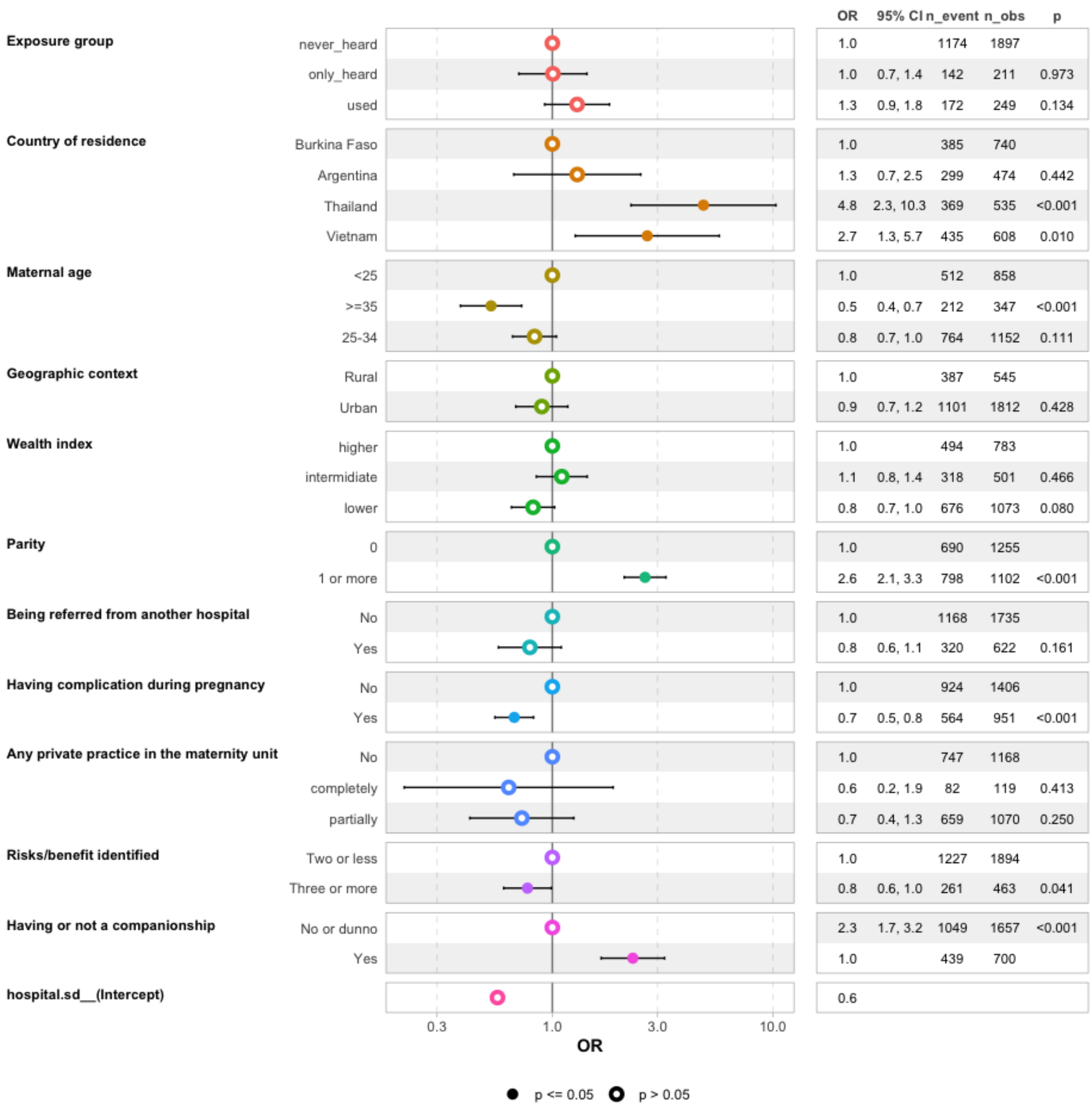

Figure S10: Multilevel logistic regression model for the association of matched preference and actual mode of birth with exposure, adjusted on selected confounders

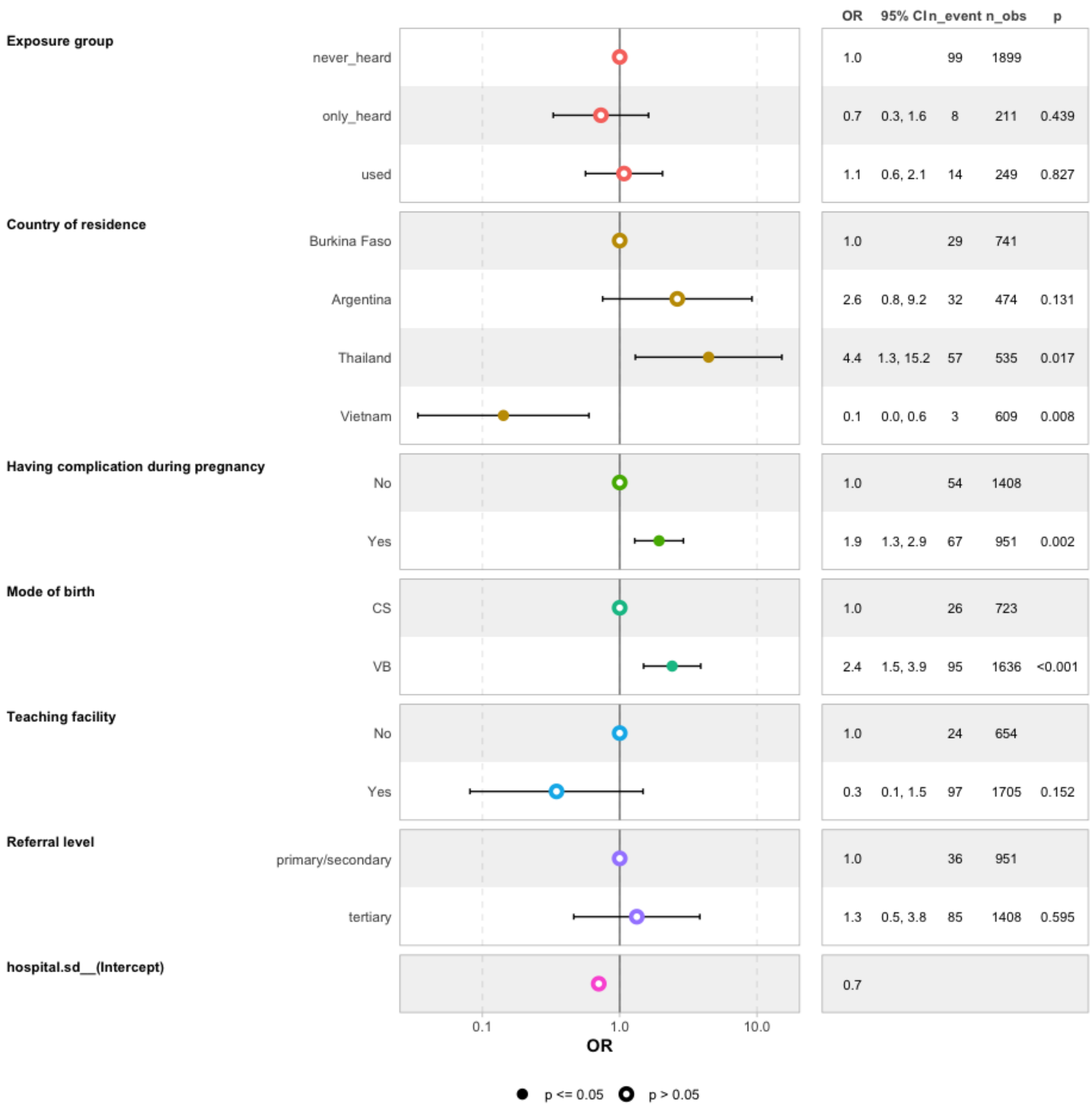

Figure S11: Multilevel logistic regression model for the association of women with postpartum haemorrhage treatment with exposure, adjusted on selected confounders

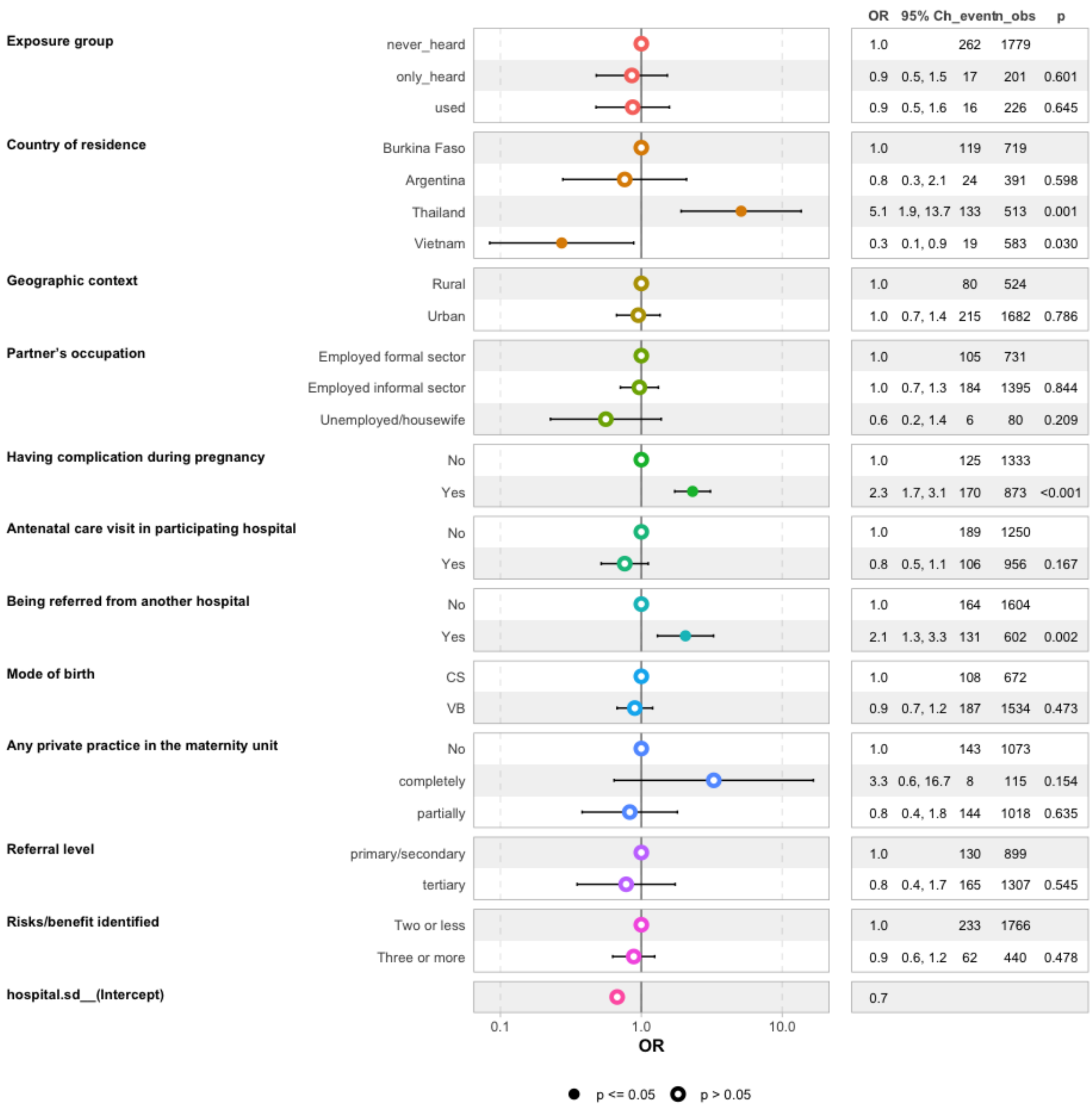

Figure S12: Multilevel logistic regression model for the association of neonatal ICU admission with exposure, adjusted on selected confounders

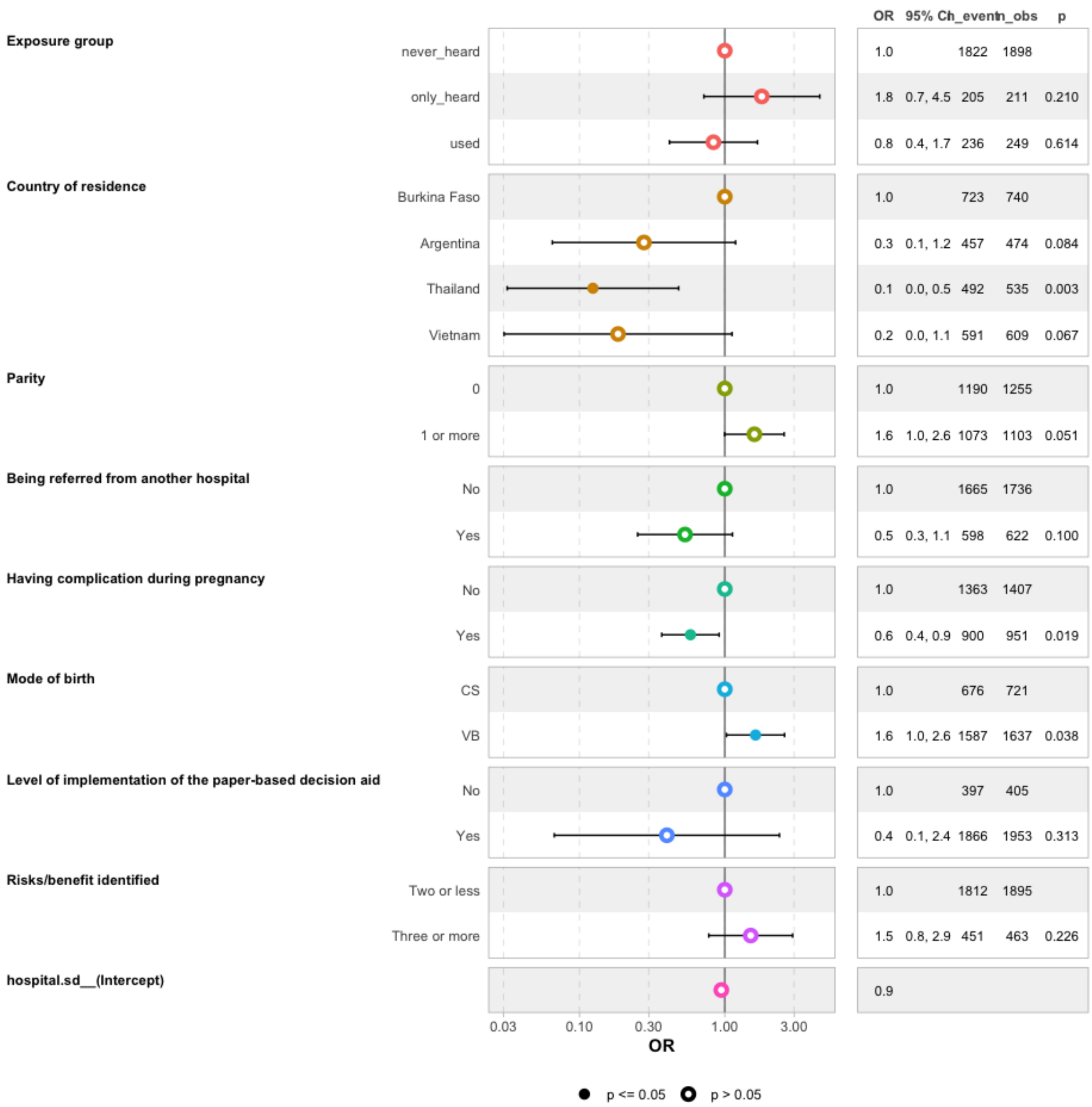

Figure S13: Multilevel logistic regression model for the association of preference for breastfeeding with exposure, adjusted on selected confounders

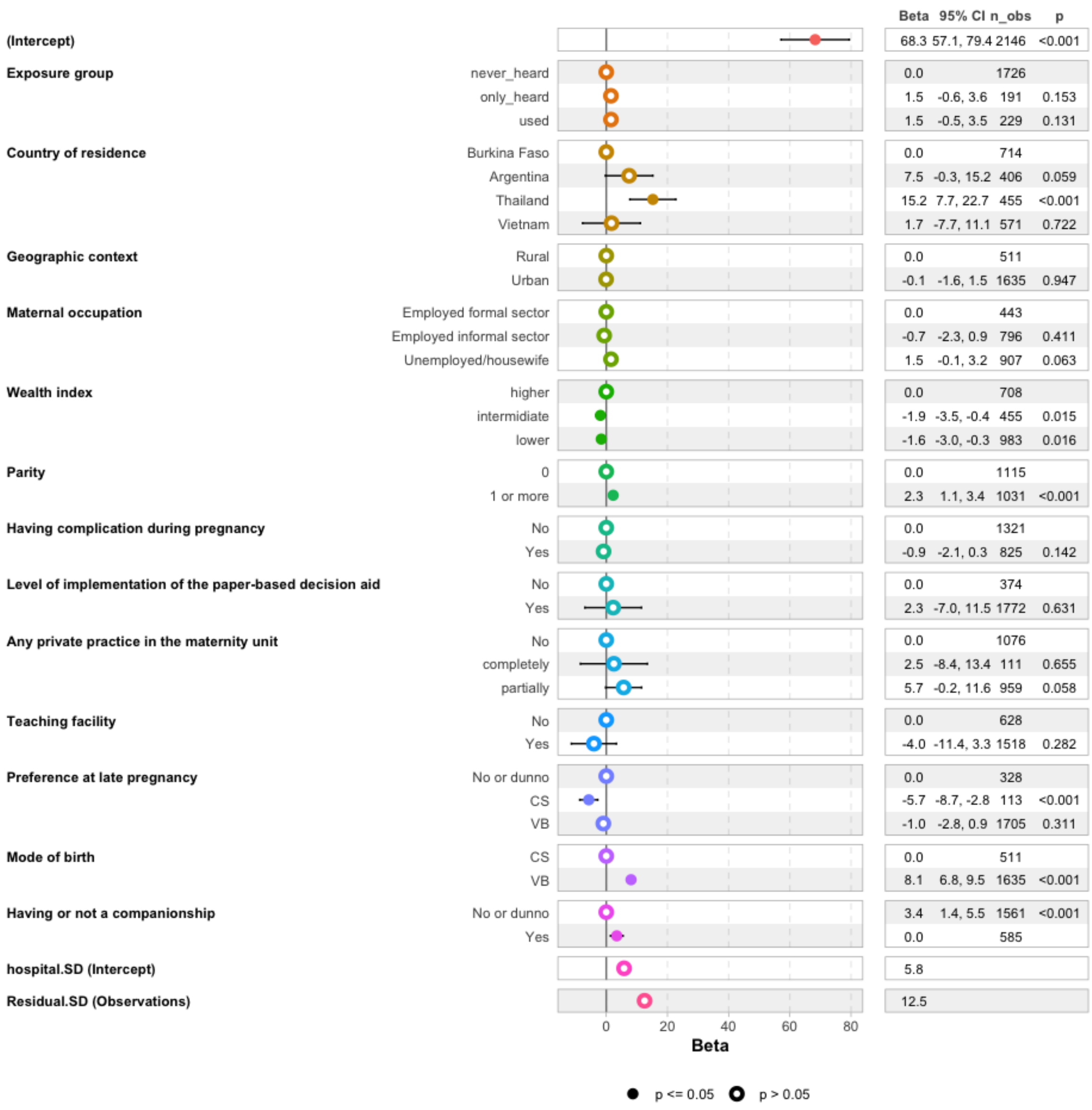

Figure S14: Multilevel linear regression model for the association of BES-emotional support score with exposure, adjusted on selected confounders

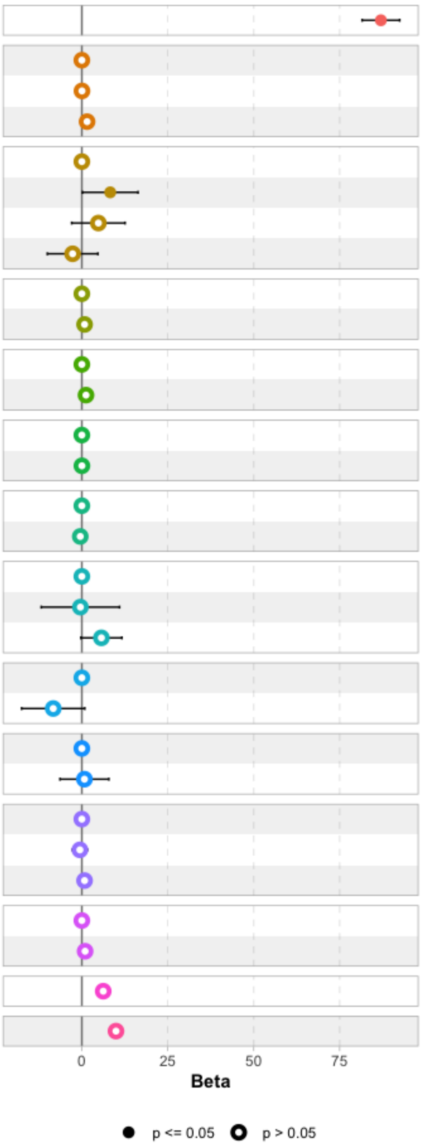

		Beta	95% CI	n_obs	p
**(Intercept)**		87.0	81.5, 92.5	2243	<0.001
**Exposure group**	never_heard	0.0		1798	
	only_heard	0.0	-1.6, 1.6	203	0.965
	used	1.5	0.0, 3.1	242	0.052
**Country of residence**	Burkina Faso	0.0		660	
	Argentina	8.3	0.2, 16.4	458	0.046
	Thailand	4.8	-2.9, 12.6	532	0.224
	Vietnam	-2.7	-10.0, 4.7	593	0.476
**Time to get to hospital**	< 30 minutes	0.0		1174	
	> 30 minutes	0.8	-0.1, 1.7	1069	0.088
**Number of antenatal care visits**	0-3	0.0		269	
	4 or more	1.2	-0.2, 2.6	1974	0.088
**Being referred from another hospital**	No	0.0		1687	
	Yes	0.0	-1.5, 1.5	556	0.978
**Having complication during pregnancy**	No	0.0		1336	
	Yes	-0.5	-1.4, 0.5	907	0.327
**Any private practice in the maternity unit**	No	0.0		1103	
	completely	-0.4	-11.8, 11.0	116	0.942
	partially	5.7	-0.3, 11.7	1024	0.062
**Teaching facility**	No	0.0		603	
	Yes	-8.3	-17.5, 0.9	1640	0.076
**Referral level**	primary/secondary	0.0		885	
	tertiary	0.8	-6.4, 7.9	1358	0.835
**Preference at late pregnancy**	No or dunno	0.0		315	
	CS	-0.6	-2.7, 1.6	196	0.609
	VB	0.8	-0.9, 2.5	1732	0.351
**Matched preferred and actual MOB**	No	0.0		813	
	Yes	0.9	-0.1, 2.0	1430	0.075
**hospital.SD (Intercept)**		6.2			
**Residual.SD (Observations)**		9.9			

Figure S15: Multilevel linear regression model for the association of BES-respect and dignity score with exposure, adjusted on selected confounders

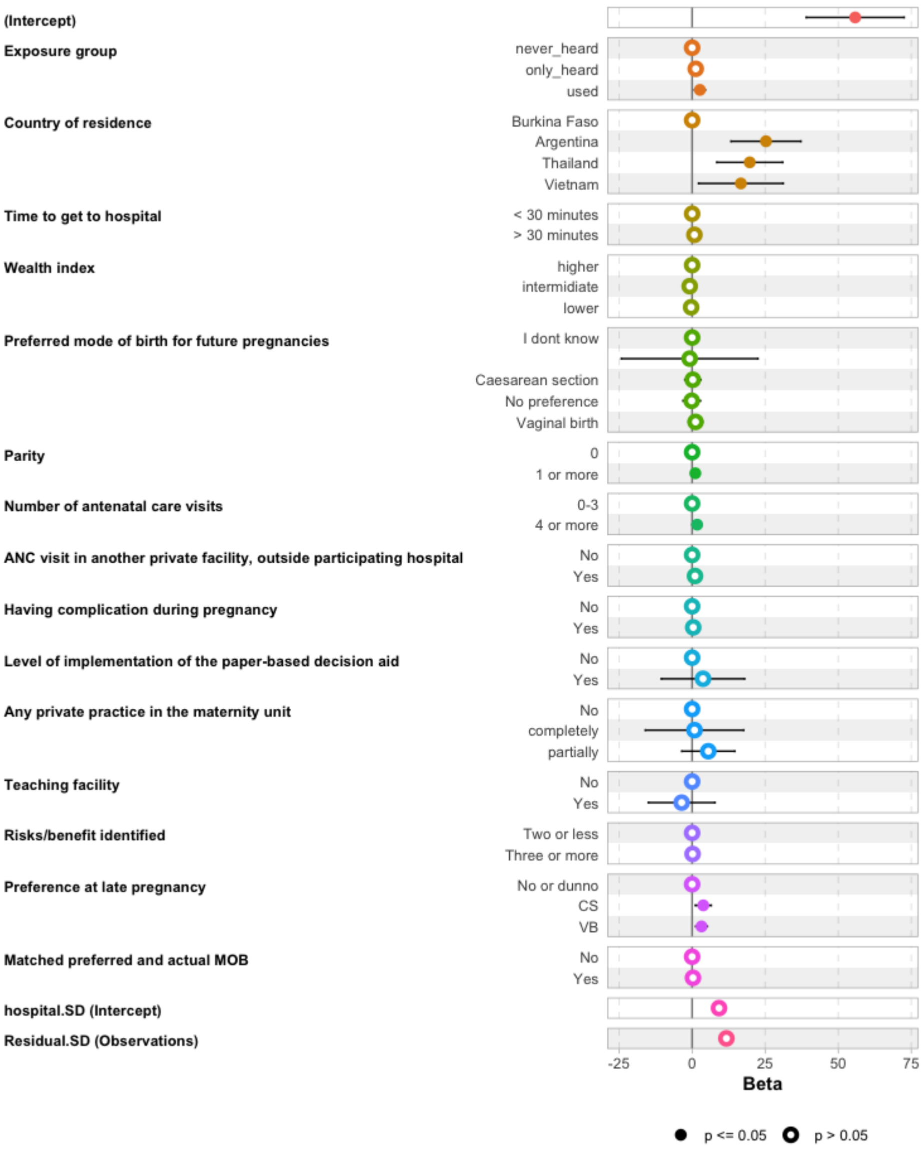

		Beta	95% CI	n_obs	p
(Intercept)		55.8	39.0, 72.5	2239	<0.001
Exposure group	never_heard	0.0		1793	
	only_heard	1.2	-0.7, 3.1	203	0.203
	used	2.7	0.8, 4.5	243	0.004
Country of residence	Burkina Faso	0.0		656	
	Argentina	25.3	13.3, 37.2	459	<0.001
	Thailand	19.7	8.4, 31.0	532	<0.001
	Vietnam	16.7	2.2, 31.2	592	0.024
Time to get to hospital	< 30 minutes	0.0		1172	
	> 30 minutes	0.8	-0.3, 1.8	1067	0.153
Wealth index	higher	0.0		751	
	intermidiate	-0.8	-2.2, 0.6	482	0.243
	lower	-0.4	-1.6, 0.9	1006	0.566
Preferred mode of birth for future pregnancies	I dont know	-0.8	-24.2, 22.5	1	0.944
		0.0		106	
	Caesarean section	0.2	-2.5, 2.9	331	0.872
	No preference	-0.2	-3.1, 2.8	163	0.921
	Vaginal birth	1.2	-1.2, 3.5	1638	0.335
Parity	0	0.0		1187	
	1 or more	1.1	0.1, 2.1	1052	0.038
Number of antenatal care visits	0-3	0.0		268	
	4 or more	1.7	0.1, 3.4	1971	0.041
ANC visit in another private facility, outside participating hospital	No	0.0		1484	
	Yes	1.0	-0.3, 2.3	755	0.137
Having complication during pregnancy	No	0.0		1333	
	Yes	0.4	-0.7, 1.4	906	0.499
Level of implementation of the paper-based decision aid	No	0.0		389	
	Yes	3.7	-10.5, 18.0	1850	0.609
Any private practice in the maternity unit	No	0.0		1101	
	completely	0.8	-16.0, 17.7	116	0.924
	partially	5.5	-3.5, 14.6	1022	0.232
Teaching facility	No	0.0		600	
	Yes	-3.6	-15.0, 7.8	1639	0.538
Risks/benefit identified	Two or less	0.0		1811	
	Three or more	0.2	-1.2, 1.5	428	0.820
Preference at late pregnancy	No or dunno	0.0		314	
	CS	3.8	1.1, 6.5	196	0.006
	VB	3.2	1.2, 5.2	1729	0.001
Matched preferred and actual MOB	No	0.0		810	
	Yes	0.3	-1.0, 1.6	1429	0.662
hospital.SD (Intercept)		9.2			
Residual.SD (Observations)		11.7			

Figure S16: Multilevel linear regression model for the association of BES-communication score with exposure, adjusted on selected confounders

		Beta	95% CI	n_obs	p
**(Intercept)**		72.7	61.7, 83.7	2238	<0.001
**Exposure group**	never_heard	0.0		1793	
	only_heard	0.8	-0.6, 2.2	203	0.259
	used	1.9	0.5, 3.3	242	0.006
**Country of residence**	Burkina Faso	0.0		656	
	Argentina	12.8	5.0, 20.7	458	0.001
	Thailand	11.3	3.9, 18.6	532	0.003
	Vietnam	4.8	-4.7, 14.3	592	0.322
**Geographic context**	Rural	0.0		501	
	Urban	0.0	-1.1, 1.1	1737	0.995
**Time to get to hospital**	< 30 minutes	0.0		1171	
	> 30 minutes	0.2	-0.6, 1.1	1067	0.567
**Wealth index**	higher	0.0		750	
	intermidiate	-0.8	-1.9, 0.2	482	0.123
	lower	-0.4	-1.3, 0.5	1006	0.423
**Parity**	0	0.0		1187	
	1 or more	1.1	0.3, 1.9	1051	0.006
**Number of antenatal care visits**	0-3	0.0		268	
	4 or more	1.4	0.2, 2.6	1970	0.027
**Having complication during pregnancy**	No	0.0		1332	
	Yes	-0.4	-1.2, 0.4	906	0.335
**Level of implementation of the paper-based decision aid**	No	0.0		389	
	Yes	2.4	-7.0, 11.7	1849	0.618
**Any private practice in the maternity unit**	No	0.0		1100	
	completely	0.5	-10.6, 11.5	116	0.933
	partially	5.6	-0.3, 11.6	1022	0.064
**Teaching facility**	No	0.0		600	
	Yes	-5.3	-12.8, 2.2	1638	0.164
**Preference at late pregnancy**	No or dunno	0.0		314	
	CS	0.5	-1.7, 2.8	196	0.646
	VB	1.1	-0.5, 2.7	1728	0.175
**Mode of birth**	CS	0.0		679	
	VB	2.3	1.0, 3.5	1559	<0.001
**Matched preferred and actual MOB**	No	0.0		810	
	Yes	0.5	-0.8, 1.9	1428	0.444
**hospital.SD (Intercept)**		6.0			
**Residual.SD (Observations)**		8.8			

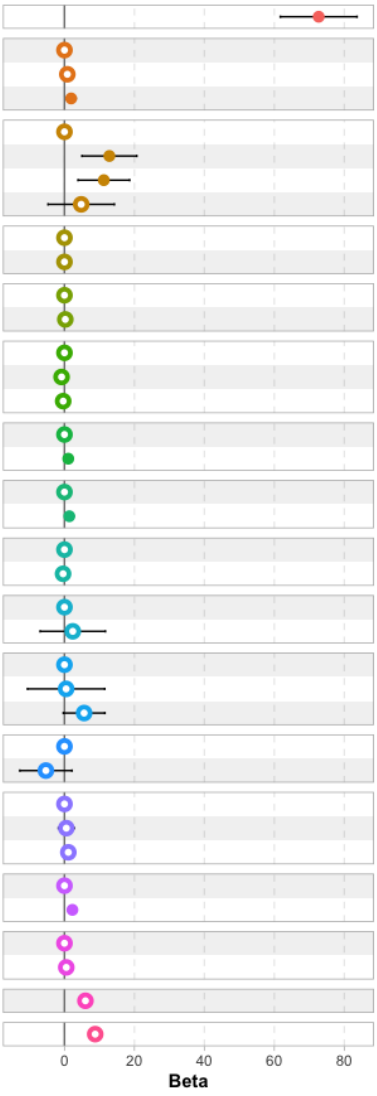

● p <= 0.05 ○ p > 0.05

Figure S17: Multilevel linear regression model for the association of overall birth experience and satisfaction score with exposure, adjusted on selected confounders
